# New Nitrogen, Sulfur-, and Selenium-Donating Ligands Derived from Chiral Pyridine Amino Alcohols. Synthesis and Catalytic Activity in Asymmetric Allylic Alkylation

**DOI:** 10.3390/molecules26123493

**Published:** 2021-06-08

**Authors:** Marzena Wosińska-Hrydczuk, Jacek Skarżewski

**Affiliations:** Chair of Organic and Medicinal Chemistry, Faculty of Chemistry, Wrocław University of Science and Technology, Wyb. Wyspiańskiego 27, 50-370 Wrocław, Poland; marzena.wosinska@pwr.edu.pl

**Keywords:** chiral pyridine compounds, thioethers, selenoethers, chiral diamine Schiff bases, chiral ligands, asymmetric allylic alkylation

## Abstract

Although many chiral ligands for asymmetric catalysis have been developed, there is still a need for new structures allowing the modular approach. Recently, easy synthesis of chiral pyridine-containing β-amino alcohols has been elaborated by opening respective epoxides with enantiomeric 1-phenylethylamine. This paper reports the synthetic transformation of β-amino alcohols into the new complexing pyridine-containing seleno- and thioethers. The amino alcohols were effectively converted to cyclic sulfonamidates, which were reacted with thiolates or phenyl selenide nucleophile. The reaction was diastereoselective, and its outcome depended on the configuration at the substitution center. The problem was discussed considering DFT optimized structures of both diastereomeric sulfonamidates. New amino-aldimine ligands were also synthesized from chiral pyridine-containing diamines. Nine new chiral ligands were tested in the Tsuji-Trost allylic alkylation resulting in the enantiomerically enriched product in up to 75% ee. The observed stereochemical induction agrees with the prevailing nucleophilic attack at the allylic carbon laying opposite to the complexing nitrogen of pyridine in η^3^-allylic intermediate complexes.

## 1. Introduction

**Figure 1:**
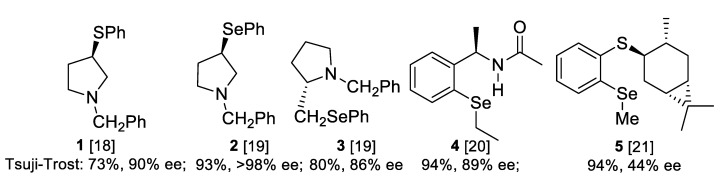
Examples of nitrogen-sulfur and selenium chiral ligands.

Catalytic properties of metal complexes used in asymmetric reactions depend on the coordinated metal and the topology of the chiral ligand. Also, an electronic character of the donating (metal-binding) groups is of primary importance [1,2]. Their σ-donating and π-accepting (back-bonding) properties influence the reactivity of the key catalytic complexes. The chiral sulfur [3,4,5] and selenium [6,7], in addition to the foremost used phosphorus and nitrogen [1,2,8] donating ligands play an important role in asymmetric catalysis. In particular, the asymmetric allylic alkylations catalyzed by *Pd* (the Tsuji-Trost reaction) [9,10,11,12,13,14,15,16,17] have been carried out in the presence of the ligands with sulfide, selenide, and diselenide groups, as exemplified by chiral ligands **1–5**, [18,19,20,21] (Figure 1). Though, the literature reports a moderate number of similar catalysts used in the Tsuji-Trost reaction [22,23,24]. In particular, the interplay between the pyridine nitrogen and sulfide- or selenide-donating site seems to deserve further investigation. Thus, the direct enantioselective transformation of the easily available chiral β-amino alcohols containing pyridine unit [25] to the new *S* and *Se* derivatives was attempted. However, the nucleophilic substitution of the corresponding oxophosphonium-activated hydroxyls resulted in the epimerization at the substitution center. In other cases, the respective internal reaction led to the corresponding aziridines. Thus, to achieve our task, we adopted the synthetic procedure via cyclic sulfonamidates. Interestingly, also outcomes of these reactions depended on the stereochemistry of the epimeric substrates. For comparison, the previously obtained chiral diamines [26] were transformed into amino-aldimine derivatives, analogous to the well-known salen-type ligands. The obtained chiral ligands were examined in the palladium-catalyzed Tsuji-Trost reaction, giving up to 75% ee. The absolute configuration of the product explains the preferred direction of the nucleophilic attack and suggests a stronger π-accepting character of pyridine nitrogen over chalcogen ethers.

## 2. Results and Discussion

### 2.1. Synthesis of Sulfur and Selenium Derivatives

**Scheme 1:**
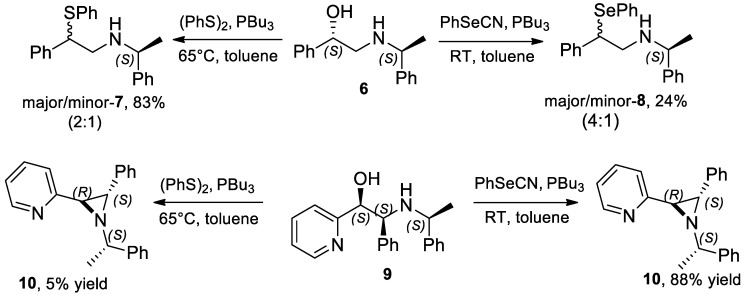
Attempted synthesis of chiral sulfur and selenium derivatives.

To prepare chiral *N*, *S* donating ligands, we applied the Hata reaction conditions (Bu_3_P, (PhS)_2_, in toluene under Ar, in a sealed tube at 65 °C) [27,28]. When the reaction was run with the amino alcohol (1*S*,1′*S*)-**6**, the respective phenylsulphanyl derivative **7** resulted in good yield (Scheme 1), but the product was obtained as a mixture of both diastereomers (ca. 2:1). Though, when we used the pyridine amino alcohol (1*S*,2*S*,1′*S*)-**9,** we couldn’t obtain the desired sulfur-containing compounds. The reaction resulted in a very small amount of the corresponding aziridines only (Scheme 1). We also used the Grieco procedure (Bu_3_P, PhSeCN, in toluene under *Ar*, at 25 °C) [28,29] to obtain the corresponding chiral selenide (Scheme 1). The hydroxyl group was again activated as the oxyphosphonium salt and reacted with the phenylselenide anion, giving a mixture of diastereomeric direct substitution products **8** (4:1). However, when we used amino alcohol (1*S*,2*S*,1′*S*)-**9** in this reaction, we obtained aziridine (2*R*,3*S*,1′*S*)-**10** in 88% yield instead the selenium compound.

**Figure 2:**
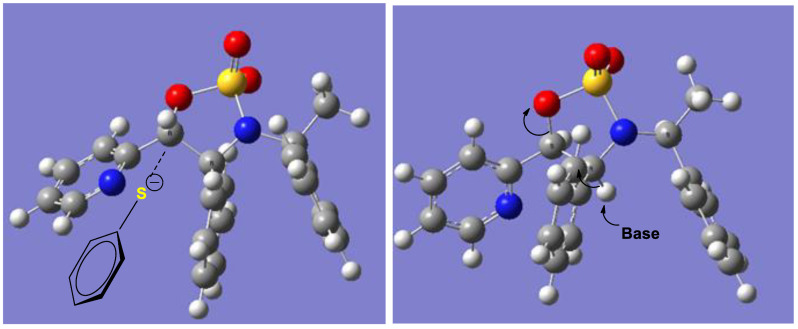
Molecular geometry of lowest energy structures optimized at the DFT/B3LYP/CC-pVDZ level for (4*R*,5*R*,1′*S*)-**13** (**left**) and (4*S*,5S,1′*S*)-**13** (**right**).

**Scheme 2:**
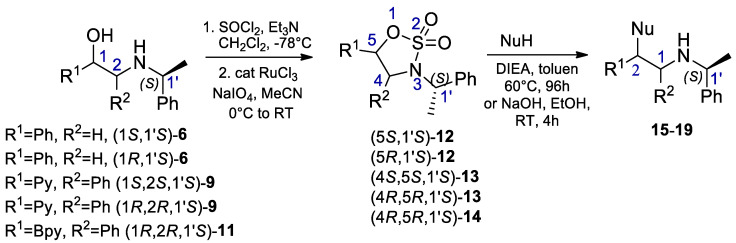
Syntheses of *S* and *Se* compounds via nucleophilic substitution of sulfonamidates.

Due to the lack of selectivity in the method described above, the procedure was changed. To develop the enantioselective transformation of chiral β-amino alcohols to new phenylsulfanyl derivatives, we have adopted the synthetic procedure via cyclic sulfonamidates, which were obtained earlier in our laboratory [26]. The cyclic amidates could be prepared by a simple reaction with thionyl chloride followed by the in situ oxidation of the product. So the corresponding sulfonamides were used as the starting material in the reaction with a sulfur nucleophile in the presence of diisopropylethylamine (DIEA). The reaction was carried out in toluene for 4 days at 60 °C to give the corresponding products **15**–**16** in yields 5–49% (Scheme 2, Method 1) (Table 1). The slow progress of the reaction was observed for the pyridine derivatives; therefore, the reaction conditions were changed. Thioles or selenide derivatives were mixed with cyclic sulfonamidates in the presence of 1.5 eq of NaOH suspension in ethanol, which gave products **16**–**19** in higher yields. This procedure allowed to shorten the reaction time from 4 days to 4 h—Method 2 (Table 1). Interestingly, when we used (4*S*,5*S*,1′*S*)-**13**, the significant drop in yield was observed in Method 1, the product (1*S*, 2*R*,1′*S*)-**16** was only formed in just 5% yield (identified by ^1^H NMR), and the starting material could be recovered from the reaction mixture. Method 2 resulted in an elimination reaction, and the inseparable mixture containing enamine product was obtained (identified by ^1^H NMR, see SI: Figure S25). To explain these phenomena, we modeled both epimeric cyclic sulfonamidates **13** using the DFT geometry optimization at the B3LYP/CC-pVDZ level of theory utilizing Gaussian code (Figure 2) [30]. The obtained structures clarify the observed diastereoselectivity. Thus, for (4*R*,5*R*,1′*S*)-**13**, the observed nucleophilic attack on the C-5 could take place, while for (4*S*,5*S*,1′*S*)-**13**, the respective substitution product was formed in a very small amount (5% only), because of the steric hindrance (Figure 2). Moreover, the trans-location of the leaving group at the C-5 and the hydrogen atom at C-4 in the reaction in the presence of NaOH caused the observed elimination.

**Figure 3:**
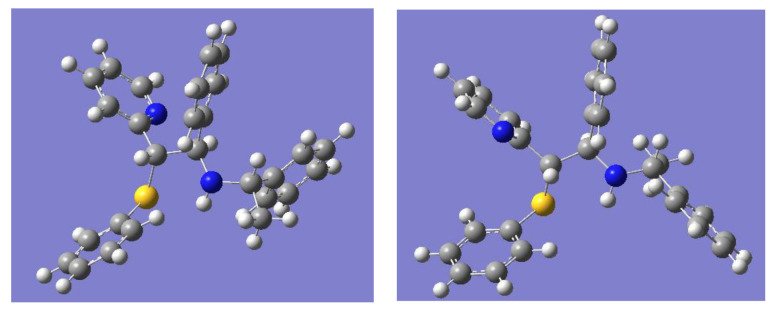
Molecular geometry of lowest energy structures optimized at the DFT/B3LYP/CC-pVDZ level for. (1*R*,2*S*,1′*S*)-**16** (**left**) and (1*S*,2*R*,1′*S*)-**16** (**right**).

The obtained phenylsulfanyl derivatives (1*R*, 2*S*,1′*S*)-**16** and (1*S*, 2*R*,1′*S*)-**16** were subjected to the DFT geometry optimization at the B3LYP/CC-pVDZ level of theory with Gaussian code [30] (Figure 3). GIAO isotropic shielding values were calculated and converted to chemical shifts for the most stable conformations using linear scaling factors [31]. The calculated δ values were compared with the experimental data for the very well-resolved aliphatic region. They showed a good qualitative agreement, which allowed confirming stereochemistry at the substitution center for both diastereomers (Figure 3 and SI, Table S1).

### 2.2. Synthesis of Nitrogen Derivatives (Chiral Schiff Base)

We have synthesized a library of the amino-aldimine ligands from chiral diamines [26]. Although the Schiff bases have been extensively studied [32], there is still a group of unsymmetrical ligands with unexplored catalytic applications. For this reason, we obtained appropriate derivatives containing pyridine fragments. The reaction was run in toluene with salicylaldehydes in the presence of molecular sieves 4 Å. The products **22**–**24** were obtained in 60–70% yield (Table 2).

### 2.3. Application of Chiral S, Se, and N Derivatives in the Tsuji-Trost Reaction

The obtained compounds (2*R*,1′*S*)-**15**, (1*R*,2*S*,1′S)-**16**, (1*R*,2*S*,1′S)-**17**, (1*R*,2*S*,1′S)-**18**, (1*R*,2*S*,1′*S*)-**19**, (1*S*,2*R*,1′*S*)-**22**, (2*R*,1′*S*)-**23** and (2*R*,1′*S*)-**24** were tested as chiral *Pd*-complexing ligands in the Tsuji-Trost reaction. The model reaction of dimethyl malonate with *rac*-1,3-diphenyl-2-propenyl acetate in dichloromethane was carried out in the presence of *N*,*O*-bis(trimethylsilyl)acetamide (BSA), CH_3_COOK, and dimer of allylpalladium chloride with 10 mol% chiral ligands under argon atmosphere for 24 at room temperature. The best catalytic effect of 58% ee for the *S* enantiomer of the product was observed when ligand (1*R*, 2*S*, 1′*S*)-**16** was used (Table 3). Then, the reaction temperature was optimized for reaction with (1*R*,2*S*,1′*S*)-**16** ligand, for 0–4 °C we observed an increase in enantioselectivity to 70% (*S*), and for −18 °C–75% *ee* (*S*), respectively. Further lowering the temperature did not affect the enantiomeric excess (Table 4). The stereochemistry of the product (enantiomeric excess and configuration) was determined by HPLC, comparing the obtained results with the literature data [32]. In the absence of pyridine moiety (as for the ligands **15**), the most opposite product was formed. We also run the reaction catalyzed by (1*R*,2*S*,1′*S*)-**16** between *rac*-1,3-diphenyl-2-propenyl acetate and acetylacetone and obtained the respective product in only 14% yield, 20% *ee* for *S* enantiomer. A similar reaction with ethyl acetoacetate gave a mixture of both diastereomers (ca. 1:1) in 95% yield and 20% ee for each diastereomer.

**Scheme 3:**
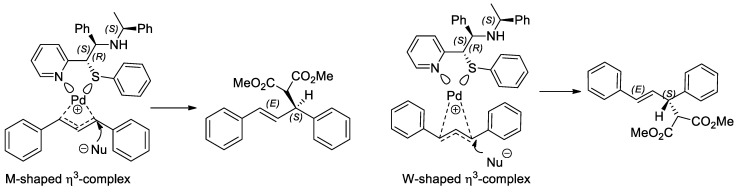
The stereochemical outcome of Tsuji-Trost reaction catalyzed by (1*R*,2*S*,1′S)-**16**—Pd complex.

The observed stereochemical outcome of the (1*R*, 2*S*,1′*S*)-**16** catalyzed allylic alkylation, i.e., formation mainly of the (*S*)-configured product, can be accounted for the preferred nucleophilic attack at the trans position [33] of the allylic part of the intermediate η^3^-allylpalladium complex with (1*R*,2*S*,1′S)-**16** (Scheme 3). The same direction of stereochemical induction (50% ee, (*S*)-alkylation product) was observed in the reaction catalyzed by the analogues selenium-containing ligand (1*R*,2*S*,1′*S*)-**19**.

Thus, for both M- and W-shaped η^3^-allylic intermediate complexes, the nucleophilic addition led to the obtained (*S*)-product. Generally, the enantioselectivity in the Tsuji-Trost reaction depends on both, steric and electronic properties of the η^3^ palladium allylic complexes [9,10,11,12,13,14,15,16,17]. An attacking nucleophile favorably approaches the complexed allylic system from the site opposite to the more π-accepting ligand center. Thus, in the case of heterobidentate *N* (sp^2^)—chalcogen ether ligands, the nucleophile approaches trans to the more π-accepting imine donor (here: pyridine nitrogen) [33,34]. For strongly σ-donating *N* (sp^3^) and weakly π-accepting chalcogen atoms, the attack trans to chalcogen is generally favored [35,36]. This last tendency may be responsible for the observed stereochemical result of the reaction catalyzed by (2*S*,1′*S*)-**15**. Also, the imino-amines lacking pyridine moiety (**23** and **24**) gave mostly the Tsuji-Trost product of (*R*)-configuration, while the respective pyridine derivative (1*S*,2*R*,1′*S*)-**22** afforded (*S*)-product with considerable ee.

## 3. Materials and Methods

### 3.1. General

Solvents were distilled, and other reagents were used as received. Reactions were monitored by thin-layer chromatography (TLC) on silica gel 60 F-254 precoated plates, and spots were visualized with a UV lamp (A.Krüss Optronic GmbH, Hamburg, Germany) and/or Dragendorff reagent. Separation of products by chromatography was carried out on silica gel 60 (230–400 mesh). Observed rotations at 589 nm were measured using an Optical Activity Ltd. Model AA-5 automatic polarimeter (Huntington, UK). ^1^H and ^13^C NMR spectra (400, 600 MHz, and 100, 151 MHz, respectively) were collected on Jeol 400 yh and Bruker Avance II 600 instruments (Karlsruhe, Germany). The spectra were recorded in CDCl_3_ referenced to the respective residual signals of the solvent. Chemical shifts are given in parts per million (ppm) and coupling constants (*J*) are in Hertz (Hz). High-resolution mass spectra were recorded using electrospray ionization on Waters LCT Premier XE TOF instrument (Milford, MA, USA). Melting points were determined using a Boëtius hotstage apparatus (PHMK VEB Analytic, Dresden, Germany). The enantiomeric ratios of the samples were determined by chiral high-performance liquid chromatography (HPLC) measurements (Thermo Fisher Scientific, Waltham, MA, USA) using Chiracel ADH chiral column.

### 3.2. General Procedure for the Synthesis of Cyclic Sulfonamidates

The synthesis of S,S-dioxides was performed according to a modified literature procedure [26,37,38]. To a solution of amino alcohol **6**, **9** or **11** (1 mmol) and triethylamine (3 mmol, 0.42 mL) in dry dichloromethane (3.5 mL) was added a solution of thionyl chloride (0.8 mmol, 58 μL) in dry dichloromethane (0.25 mL) at −78 °C for 20 min. The mixture was stirred at −78 °C for 20 min and 0 °C for the next 20 min. The reaction mixture was partitioned between ether and water, the organic layer was washed with brine and dried over anhydrous sodium sulfate, filtered, and the filtrate was concentrated in vacuo. The residue was dissolved in acetonitrile (4 mL), cooled to 0 °C, and NaIO_4_ (1.2 mmol, 257 mg), RuCl_3_·3H_2_O (ca. 2 mg), and water (4 mL) were added. The reaction mixture was stirred at room temperature for 1 h and then extracted 3 × Et_2_O. The combined organic extracts were washed with brine and dried over sodium sulfate. The residue was purified by column chromatography (SiO_2_, 10% AcOEt in hexane) to provide the cyclic sulfamidate. The same eluent was used for TLC; the respective *R*_f_ value is given below.

^1^H and ^13^C NMR spectra for (5*S*,1′*S*)-**12**, (4*R*,5*R*,1′*S*)-**13**, (4*S*,5*S*,1′*S*)-**13** are in agreement with the reported ones [26].

(5*R*)-Phenyl-3-(1′S-phenylethyl)-1,2,3-oxathiazolidine-2,2-dioxide [(5*R*,1′*S*)-**12**]

Colorless oil, 150 mg, 46% yield, ${\left[\alpha \right]}_{D}^{20}$ = 25 (c = 0.49 CHCl_3_), ^1^H NMR (400 MHz, CDCl_3_) δ: 7.41–7.30 (m, 10H), 5.51 (t, *J* = 7.3 Hz, 1H), 4.61 (q, *J* = 6.7 Hz, 1H), 3.54 (dd, *J* = 9.8, 6.7 Hz, 1H), 3.40 (dd, *J* = 9.8, 7.6 Hz, 1H), 1.70 (d, *J* = 6.7 Hz, 3H); ^13^C NMR (400 MHz, CDCl_3_) δ: 140.0, 135.5, 129.8, 129.1, 128.9, 128.5, 127.2, 126.5, 80.4, 57.1, 52.6, 19.2, HR-MS (ESI) [C_16_H_17_NO_3_S + Na]^+^ requires 326.0822; found 326.0826

(4*R*,5*R*)-4-Phenyl-5-(2,2′-bipyrid-6-yl)-3-(1′S-phenylethyl)- 1,2,3-oxathiazolidine-2,2-dioxide [(4*R*,5*R,*1′*S*)-**14**]

Yellow oil, 259 mg, 50% yield, ${\left[\alpha \right]}_{D}^{20}$ = 60 (*c* = 0.78 CHCl_3_);), *R*_f_ = 0.13; ^1^H NMR (400 MHz, CDCl_3_) δ: 8.63–8.61 (m, 1H), 8.18–8.15 (m, 1H), 8.06–8.04 (m, 1H), 7.79–7.75 (m, 1H), 7.52–7.48 (m, 1H), 7.34–7.25 (m, 6H), 7.07–6.95 (m, 6H), 6.16 (d, *J* = 6.11 Hz, 1H), 4.87 (d, *J* = 6.4 Hz, 1H), 4.37 (q, *J* = 6.7 Hz, 1H), 1.87 (d, *J* = 7.0 Hz, 3H); ^13^C NMR (101 MHz, CDCl_3_) *δ*: 155.4, 152.2, 149.3, 140.7, 137.4, 137.0, 135.9, 133.5, 128.9, 128.7, 128.4, 128.3, 128.2, 127.3, 124.0, 121.1, 120.7, 120.5, 82.6, 67.3, 57.0, 20.2; HR-MS (ESI) [C_26_H_23_N_3_O_3_S + H]^+^ requires 458.1533; found 458.1535

### 3.3. General Procedure for the Synthesis of S and Se Derivatives

Method 1: Sulfonamide (0.2 mmol) was dissolved in toluene (1 mL), then diisopropylethylamine (0.3 mmol, 0.055 mL) and thiophenol (0.25 mmol, 0.024 mL) were added. The mixture was stirred at 60 °C under argon for 4 days. The crude mixture was then applied to a chromatography column (SiO_2_, 30% AcOEt in hexane) for product isolation. The same eluent was used for TLC; the respective *R*_f_ values are given below.

Method 2: Sulfonamide (0.2 mmol or 0.5 mmol for reaction with *Se* derivatives) was dissolved in EtOH (0.5 mL), then a suspension of NaOH (15 mg) in EtOH (1 mL) and the appropriate sulfur or selenium derivative (0.25 mmol or 0.55 mmol for *Se* derivatives) were added. The mixture was stirred at 60 °C under argon for 4 h. Then, ethanol was evaporated, 1 mL of H_2_O was added, followed by extraction with 3 × 5 mL of Et_2_O, dried over Na_2_SO_4_. The product was isolated in the same way as in method 1.

(2*R*)-2-phenyl-*N*-((1*S*)-phenylethyl)-2-(phenylsulfanyl) ethanamine [(2*R*,1′*S*)-**15**]

Yellow oil, 33 mg, 49% yield (Method 1), ${\left[\alpha \right]}_{D}^{20}$ = −45 (*c* = 0.82, CHCl_3_); *R*_f_ = 0.38; ^1^H NMR (400 MHz, CDCl_3_) δ: 7.33–7.14 (m, 15H), 4.31 (t, *J* = 7.3 Hz, 1H), 3.76 (q, *J* = 6.7 Hz, 1H), 2.99–2.88 (m, 2H), 1.30 (d, *J* = 6.4 Hz, 3H); ^13^C NMR (101 MHz, CDCl_3_) *δ*: 145.4, 140.7, 134.6, 132.1, 128.8, 128.6, 128.5, 128.01, 127.98, 127.5, 127.1, 126.7, 57.9, 53.6, 52.3, 24.4; HR-MS (ESI) [C_22_H_23_NS + H]^+^ requires 334.1624; found 334.1617.

(2*S*)-phenyl-*N*-((1*S*)-phenylethyl)-2-(phenylsulfanyl) ethanamine [(2*S*,1′*S*)-**15**]

White solid, 14 mg, 21% yield (Method 2), ${\left[\alpha \right]}_{D}^{20}$ = 63 (*c* = 0.32, CHCl_3_) m.p 89–91 °C; *R*_f_ = 0.25; ^1^H NMR (400 MHz, CDCl_3_) δ: 7.31–7.14 (m, 15H), 4.30 (t, *J* = 7.3 Hz, 1H), 3.76 (q, *J* = 6.7 Hz, 1H), 2.97–2.86 (m, 2H), 1.28 (d, *J* = 6.4 Hz, 3H); ^13^C NMR (101 MHz, CDCl_3_) *δ*: 145.2, 140.4, 134.6, 132.2, 128.8, 128.6, 128.5, 128.0, 127.6, 127.2, 127.1, 126.6, 56.0, 53.3, 52.1, 24.5; HR-MS (ESI) [C_22_H_23_NS + H]^+^ requires 334.1624; found 334.1627.

*N*-(1′S-phenylethyl)-(1*R*)-phenyl-(2*S*)-(phenylsulfanyl)-(2*S*)-pyridin-2-yl ethanamine [(1*R*,2*S*,1′*S*)-**16**]

Yellow oil, 48 mg, 58% yield (Method 2), ${\left[\alpha \right]}_{D}^{20}$ = −128 (*c* = 0.94 CHCl_3_); *R*_f_ = 0.45; ^1^H NMR (400 MHz, CDCl_3_) δ: 8.35–8.33 (m, 1H), 7.30–7.09 (m, 16H), 6.92–6.89 (m, 1H), 6.81 (d, *J* = 7.9 Hz, 1H), 4.46 (d, *J* = 8.3 Hz, 1H), 3.95 (d, *J* = 8.3 Hz, 1H), 3.48 (q, *J* = 6.7 Hz, 1H), 1.32 (d, *J* = 6.7 Hz, 3H); ^13^C NMR (101 MHz, CDCl_3_) *δ*: 159.9, 153.9, 149.0, 145.3, 141.0, 135.8, 135.2, 131.7, 128.8, 128.5, 128.3, 128.0, 127.2, 126.8, 126.7, 123.4, 121.7, 63.5, 62.8, 55.1, 25.1; HR-MS (ESI) [C_27_H_26_N_2_S + H]^+^ requires 411.1889, found 411.1894.

*N*-(1′S-phenylethyl)-(1*S*)-phenyl-(2*R*)-(phenylsulfanyl)-(2R)-pyridin-2-yl ethanamine [(1*S*,2*R*,1′*S*)-**16**]

Yellow oil, 4 mg, 5% yield (Method 1), *R*_f_ = 0.36, ^1^H NMR (400 MHz, CDCl_3_) δ: 8.48–8.46 (m, 1H), 7.33–7.07 (m, 16H), 6.98–6.95 (m, 1H), 6.83 (d, *J* = 7.9 Hz, 1H), 4.53 (d, *J* = 8.2 Hz, 1H), 4.47 (d, *J* = 8.2 Hz, 1H), 3.68 (q, *J* = 6.4 Hz, 1H), 1.35 (d, *J* = 6.4 Hz, 3H).

*N*-(1′S-phenylethyl)-(1*R*)-phenyl-(2*S*)-(benzylsulfanyl)-(2*S*)-pyridin-2-yl ethanamine [(1*R*,2*S*,1′*S*)-**17**]

Yellow oil, 40 mg, 47% yield (Method 2), ${\left[\alpha \right]}_{D}^{20}$ = −153 (*c* = 1.05, CHCl_3_); *R*_f_ = 0.14; ^1^H NMR (400 MHz, CDCl_3_) δ: 8.38–8.36 (m, 1H), 7.37–7.32 (m, 1H), 7.28–7.17 (m, 6H), 7.15–7.05 (m, 9H), 6.96–6.93 (m, 1H), 6.86 (d, *J* = 7.9 Hz, 1H), 4.01 (d, *J* = 8.2 Hz, 1H), 3.85 (d, *J* = 8.6 Hz, 1H), 3.51 (d, *J* = 2.8 Hz, 2H), 3.42 (q, *J* = 6.7 Hz, 1H), 1.28 (d, *J* = 6.7 Hz, 3H); ^13^C NMR (101 MHz, CDCl_3_) *δ*: 160.2, 148.9, 145.4, 141.3, 137.9, 135.9, 129.1, 128.5, 128.4, 128.3, 128.0, 127.1, 127.0, 126.8, 126.7, 123.5, 121.7, 63.0, 59.0, 54.9, 35.9, 25.1; HR-MS (ESI) [C_28_H_28_N_2_S + H]^+^ requires 425.2046; found 425.2054.

*N*-(1′S-phenylethyl)-(1*R*)-phenyl-(2*S*)-(phenylsulfanyl)-(2S)-(2,2′-Bipyridin-6-yl)ethanamine [(1*R*,2*S*,1′*S*)-**18**]

Yellow oil, 55 mg, 56% yield (Method 2), ${\left[\alpha \right]}_{D}^{20}$ = −132 (*c* = 1.04, CHCl_3_); *R*_f_ = 0.20; ^1^H NMR (400 MHz, CDCl_3_) δ: 8.61–8.60 (m, 1H), 8.07–8.04 (m, 1H), 7.75–7.71 (m, 1H), 7.46 (q, *J* = 7.6 Hz, 1H), 7.28–7.06 (m, 17H), 6.91 (d, *J* = 7.6 Hz, 1H), 4.50 (d, *J* = 7.6 Hz, 1H), 4.11 (d, *J* = 7.6 Hz, 1H), 3.51 (q, *J* = 6.7 Hz, 1H), 1.34 (d, *J* = 6.7 Hz, 3H); ^13^C NMR (101 MHz, CDCl_3_) *δ*: 159.0, 156.2, 155.0, 149.2, 149.0, 137.6, 136.9, 136.8, 132.0, 128.9, 128.7, 128.4, 128.0, 127.2, 126.9, 126.8, 126.7, 126.3, 123.6, 123.2, 121.4, 118.9, 63.2, 55.1, 40.6, 24.8; HR-MS (ESI) [C_32_H_29_N_3_S + H]^+^ requires 488.2155; found 488.2165.

*N*-(1′S-phenylethyl)-(1*R*)-phenyl-(2*S*)-(phenylselenyl)-(2S)-pyridin-2-yl)ethanamine [(1*R*,2*S*,1′*S*)-**19**]

Colorless oil, 24 mg, 10% wydajności (Method 2), ${\left[\alpha \right]}_{D}^{20}$ = −112 (*c* = 0.74, CHCl_3_); ^1^H NMR (400 MHz, CDCl_3_) δ: 8.33–8.31 (m, 1H), 7.33–7.05 (16H), 6.86–6.83 (m, 1H), 6.57–6.55 (m, 1H), 4.49 (d, *J* = 8.6 Hz, 1H), 4.06 (d, *J* = 8.6 Hz, 1H), 3.48 (q, *J* = 6.7 Hz, 1H), 1.32 (d, *J* = 6.7 Hz, 3H); ^13^C NMR (101 MHz, CDCl_3_) *δ*: 160.3, 149.0, 145.3, 141.5, 135.6, 135.2, 128.8, 128.5, 128.12, 128.08, 127.5, 127.04, 126.98, 126.9, 126.7, 123.1, 121.4, 63.5, 58.9, 55.1, 25.2; ^77^Se NMR (38 MHz, CDCl_3_): δ _Se_ 416.99; HR-MS (ESI) [C_27_H_26_N_2_Se + H]^+^ requires 459.1334; found 459.1351

### 3.4. General Procedure for the Synthesis of Mono-Aldimine (Chiral Schiff Base)

To a solution of the diamine (0.15 mmol) in dry toluene (1 mL) were added a solution of the aldehyde (0.15 mmol) in the dry toluene (0.5 mL) and Activated molecular sieves 4Å under argon atmosphere. The reaction was carried out at RT for 6h, and then the crude mixture was separated by column chromatography (SiO_2_, 20% AcOEt in hexane) to give a pure product. The same eluent was used for TLC; the respective *R*_f_ values are given below.

2-(*tert*-butyl)-4-methyl-6-(*E*-(((1*S*,2*R*)-1-phenyl-1-(((*S*)-1-phenylethyl)amino)-2-(pyridin-2-yl)ethyl)imino)methyl) phenol [(1*S*,2*R*,1′*S*)-**22**]

Yellow oil, 47 mg, 70% yield, ${\left[\alpha \right]}_{D}^{20}$ = 9 (c = 1.10 CHCl_3_), ^1^H-NMR (400 MHz, CDCl_3_) δ: 8.49–8.47 (m, 1H), 8.22 (s, 1H), 7.58–7.54 (m, 1H), 7.28–7.08 (m, 13H), 7.07–6.83 (m, 1H), 4.66 (d, *J* = 6.1 Hz, 1H), 4.48 (d, *J* = 6.1 Hz, 1H), 3.67 (q, *J* = 6.4 Hz, 1H), 2.25 (s, 3H), 1.45 (s, 9H), 1.21 (d, *J* = 6.4 Hz, 3H); ^13^C-NMR (151 MHz, CDCl3) δ: 197.2, 167.7, 159.9, 158.3, 148.8, 145.4, 140.4, 137.3, 136.2, 135.5, 131.5, 131.1, 130.1, 128.5, 128.2, 126.6, 123.0, 122.2, 120.5, 118.4, 81.5, 65.0, 54.9, 34.9, 29.5, 25.3, 20.7; HR-MS (ESI) [C_33_H_37_N_3_O + H]^+^ requires 492.3009; found 492.3021

2,4-(di*tert*-butyl)-6-((*E)*-((1-((*S*)-1-phenylethyl)amino)-(2*R*)-(phenyl)ethyl)imino)methyl) phenol [(2*R*,1′*S*)-**23**]

Yellow oil, 41 mg, 60% yield, ${\left[\alpha \right]}_{D}^{20}$ = 86 (c = 1.05 CHCl_3_), *R*_f_ = 0.36, ^1^H-NMR (600 MHz, CDCl_3_) δ: 8.49 (s, 1H), 7.40–7.39 (m, 1H), 7.32–7.25 (m, 10H), 7.09–7.08 (m, 1H), 4.65–4.64 (m, 1H), 3.90–3.87 (m, 1H), 3.01–2.99 (m, 2H), 1.46 (s, 9H), 1.31 (d, *J* = 7.03 Hz, 3H), 1.29 (s, 9H); ^13^C-NMR (101 MHz, CDCl_3_) δ: 166.8, 158.1, 145.3, 141.6, 140.3, 136.8, 129.1, 128.7, 128.3, 127.5, 127.3, 127.1, 126.6, 126.3, 117.9, 73.9, 58.0, 54.7, 35.2, 34.2, 31.6, 29.5, 24.6; HR-MS (ESI) [C_31_H_40_N_2_O + H]^+^ requires 457.3213; found 457.3221

2-(*tert*-butyl)-4-methyl-6-((*E)*-((1-((*S*)-1-phenylethyl)amino)-(2*R*)-phenyl)ethyl)imino)methyl)phenol [(2*R*,1′*S*)-**24**]

Yellow oil, 41 mg, 68% yield, ${\left[\alpha \right]}_{D}^{20}$ = 81 (c = 1.06 CHCl_3_), *R*_f_ = 0.30, ^1^H-NMR (600 MHz, CDCl_3_) δ: 8.40 (s, 1H), 7.32–7.23 (m, 10H), 7.22–7.14 (m, 1H), 6.92–6.91 (m, 1H), 4.46 (t, *J* = 6.7 Hz, 1H), 3.81 (q, *J* = 6.7 Hz, 1H), 2.92 (d, *J* = 6.7 Hz, 2H), 2.27 (s, 3H), 1.45 (s, 9H), 1.31 (d, *J* = 6.7 Hz, 3H); ^13^C-NMR (101 MHz, CDCl3) δ: 166.3, 158.2, 145.3, 141.5, 137.2, 130.9, 129.9, 128.7, 128.6, 127.6, 127.1, 127.0, 126.8, 126.6, 125.4, 73.9, 57.9, 54.7, 34.9, 29.5, 24.6, 20.7; HR-MS (ESI) [C_28_H_34_N_2_O + H]^+^ requires 415.2744; found 415.2753.

### 3.5. Catalytic Reaction Procedure (Tsuji-Trost)

A solution of chiral ligand (0.01 mmol) and the allylpalladium chloride dimer (1 mg, 0.0025 mmol) was stirred in dry dichloromethane (0.4 mL) under argon atmosphere at room temperature for 15 min. Then a solution of *rac*-1,3-diphenyl-2-propenyl acetate (25 mg, 0.1 mmol) in dichloromethane (0.4 mL), dimethylmalonate (0.035 mL, 0.3 mmol), N,O-bis(trimethyl-silyl)acetamide (0.075 mL, 0.3 mmol) and anhydrous potassium acetate (0.3 mg, 0.003 mmol) were added.

The reaction was carried out at room temperature for 1–4 days (monitored by TLC) [18]. After the reaction was complete, the crude mixture was filtered by SiO_2_, and then the solvent was evaporated to give the crude product as yellow oil. Enantiomeric excess was determined using a Chiralpak AD-H column (n-hexane/isopropanol 90/10, 1.0 mL/min, 254 nm) *t*_R_ 12.4 and *t*_S_ 17.2 min. Configuration assignment was based on the literature data [32]. ^1^H-NMR (400 MHz, CDCl_3_) δ: 7.32–7.18 (m, 10 H), 6.45 (d, *J* = 15.6 Hz, 1H), 6.32 (dd, *J* = 15.9, 8.9 Hz, 1H), 4.25 (dd, *J* = 11.0, 8.6 Hz, 1H), 3.93 (d, *J* = 11.0 Hz, 1H), 3.74 (s, 3H), 3.69 (s, 3H).

## 4. Conclusions

Concluding, we successfully converted chiral pyridine-containing amino alcohols into the respective thioethers, selenoethers, and the mono-Schiff bases of the corresponding diamines. These new chiral compounds were tested in the Tsuji-Trost allylic alkylation resulting in the enantiomeric enrichment of product in up to 75%. The resulting stereochemical induction agrees with the nucleophilic attack at the allylic carbon located trans to the complexing nitrogen of pyridine in η^3^-allylic intermediate complexes.

## Figures and Tables

**Table 1:** Ring-opening reactions of cyclic sulfonamidates.

Sulfonamidates	R^1^	R^2^	NuH	Yield [%] ^a^	Product 15–19
(5*S*,1′*S*)-**12**	Ph	H	PhSH	49	(2*R*,1′*S*)-**15**
(5*R*,1′*S*)-**12**	Ph	H	PhSH	21 ^a^	(2S,1′*S*)-**15**
(4*R*,5*R*,1′*S*)-**13**	Py	Ph	PhSH	30, 58 ^a^	(1*R*,2*S*,1′*S*)-**16**
(4S,5S,1′*S*)-**13**	Py	Ph	PhSH	5 ^b^	(1*S*,2*R*,1′*S*)-**16**
(4*R*,5*R*,1′*S*)-**13**	Py	Ph	PhCH_2_SH	47 ^a^	(1*R*,2*S*,1′*S*)-**17**
(4R,5R,1′*S*)-**14**	Bpy	Ph	PhSH	56 ^a^	(1*R*,2*S*,1′*S*)-**18**
(4*R*,5*R*,1′*S*)-**13**	Py	Ph	PhSeH	10 ^a^	(1*R*,2*S*,1′*S*)-**19**

^a^ using Method 2 (1.5 eq NaOH w EtOH), ^b^ identified only by ^1^H NMR.

**Table 2:**
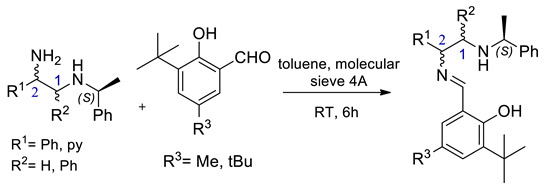
Synthesis of chiral amino-aldimines.

Chiral Diamine	R^1^	R^2^	R^3^	Yield [%]	Product
(1*S*,2*R*,1′*S*)-**20**	Py	Ph	Me	70	(1*S*,2*R*,1′*S*)-**22**
(2*R*,1′*S*)-**21**	Ph	H	tBu	60	(2*R*,1′*S*)-**23**
(2*R*,1′*S*)-**21**	Ph	H	Me	68	(2*R*,1′*S*)-**24**

**Table 3:**
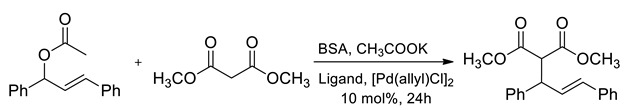
Catalytic Tsuji-Trost reaction.

Chiral Ligand	Conversion [%] ^a^	Ee [%] Configuration
(2*R*,1′*S*)-**15**	15	Rac
(2S,1′*S*)-**15**	30	31 (R)
(1*R*,2*S*,1′S)-**16**	100	58 (S)
(1*R*,2*S*,1′S)-**17**	98	26 (S)
(1*R*,2*S*,1′S)-**18**	89	13 (R)
(1*R*,2*S*,1′*S*)-**19**	70	50 (S)
(1*S*,2*R*,1′*S*)-**22**	98 ^b^	49 (S)
(2*R*,1′*S*)-**23**	66 ^b^	38 (R)
(2*R*,1′*S*)-**24**	69 ^b^	38 (R)

^a^ Calculated from ^1^H NMR, ^b^ Reaction was run for 48 h.

**Table 4:** Optimization of reaction temperature.

Chiral Ligand	Temperature [°C]	Conversion [%]	Ee [%] Configuration	Time [h]
(1*R*,2*S*,1′S)-**16**	0–4	97	70 (S)	48
(1*R*,2*S*,1′S)-**16**	−18	75	75 (S)	96
(1*R*,2*S*,1′S)-**16**	−30	5	73 (S)	10
(1*S*,2*R*,1′*S*)-**22**	−18	96	71 (S)	96

## Data Availability

All the required data are reported in the manuscript and Supplementary Materials.

## Supplementary Materials

The following are available online, Figures S1–S25: copies of ^1^H and ^13^C NMR spectra, Figures S26–S28: HPLC plots for the Tsuji-Trost reaction outcomes, Table S1: Comparison of experimental and calculated (DFT/GIAO) chemical shifts.

## Sample Availability

Samples of the compounds **12**–**19** and **22**–**24**. are available from the authors.
