# Supplementary material for: New Nitrogen, Sulfur-, and Selenium-Donating Ligands Derived from Chiral Pyridine Amino Alcohols. Synthesis and Catalytic Activity in Asymmetric Allylic Alkylation
Source: Molecules. 2021 Jun 8;26(12):3493. doi: 10.3390/molecules26123493 (PMC8228606; doi:10.3390/molecules26123493)
Supplement: Supplementary file 1 [file molecules-26-03493-s001.zip › SI.pdf]

molecules

New Nitrogen, Sulfur-, and Selenium-donating Ligands Derived from Chiral Pyridine Amino Alcohols. Synthesis and Catalytic Activity in Asymmetric Allylic Alkylation.

Marzena Wosińska-Hrydczuk and Jacek Skarżewski

Supporting information

Table of contents

1. Copies of NMR spectra
2. Catalytic Tsuji-Trost reaction- HPLC spectra
3. DFT calculation

1. Copies of NMR spectra

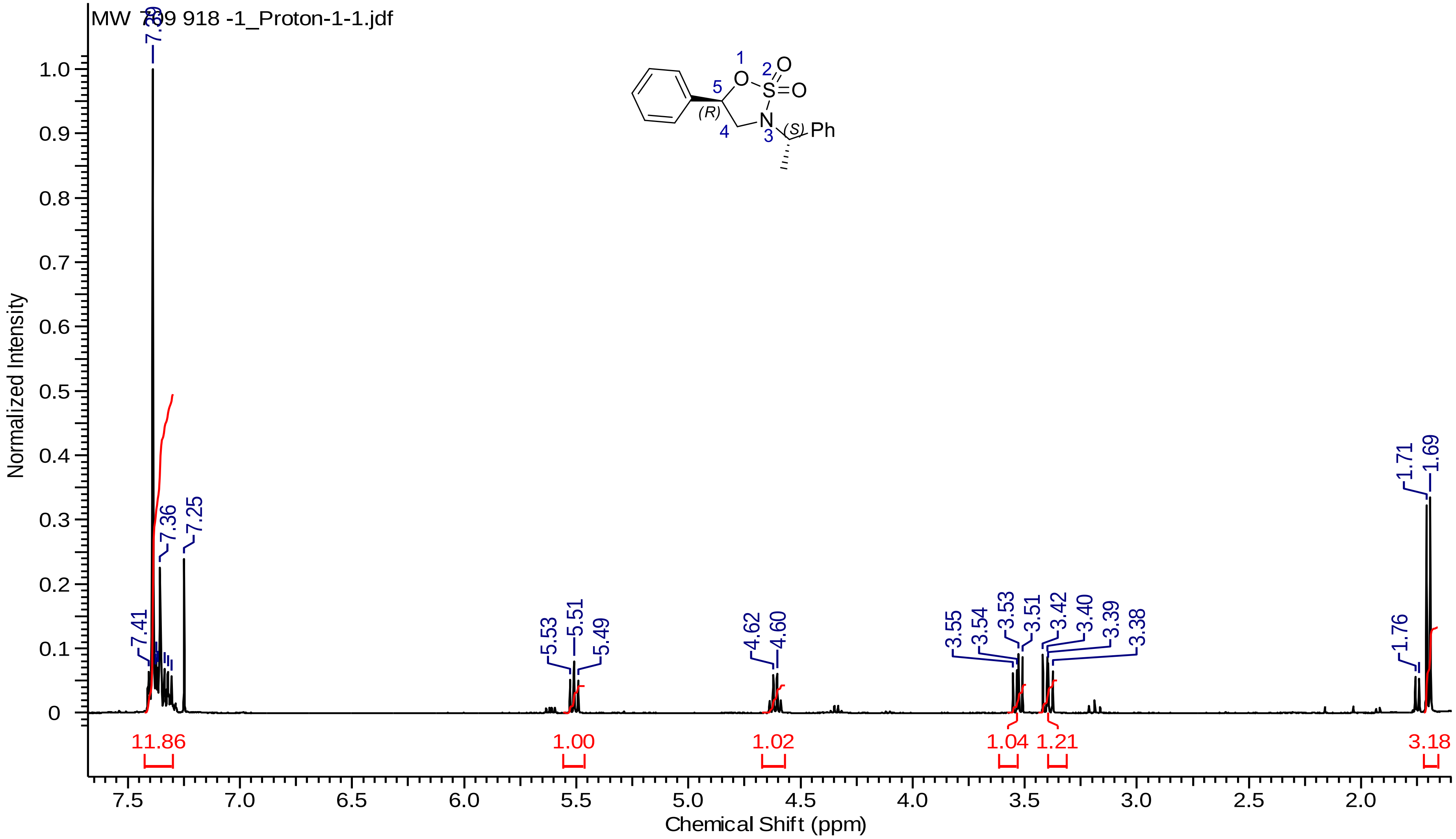

Figure S1. ^{1}H NMR spectrum (400 MHz, $CDCl_3$) for (5*R*,1'*S*)-**12**

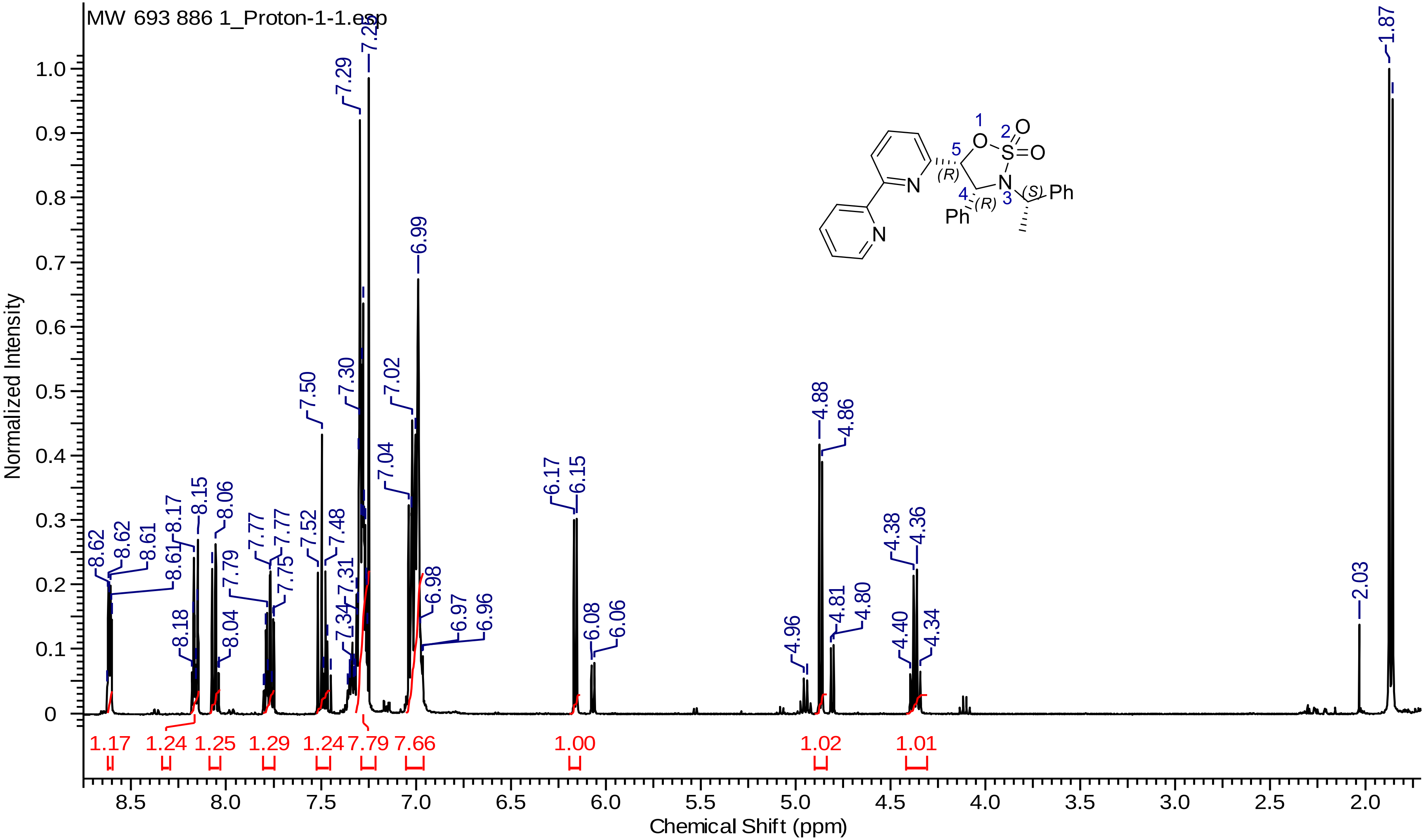

Figure S3. ^{1}H NMR spectrum (400 MHz, $CDCl_3$) for (4*R*,5*R*,1'*S*)-**14**

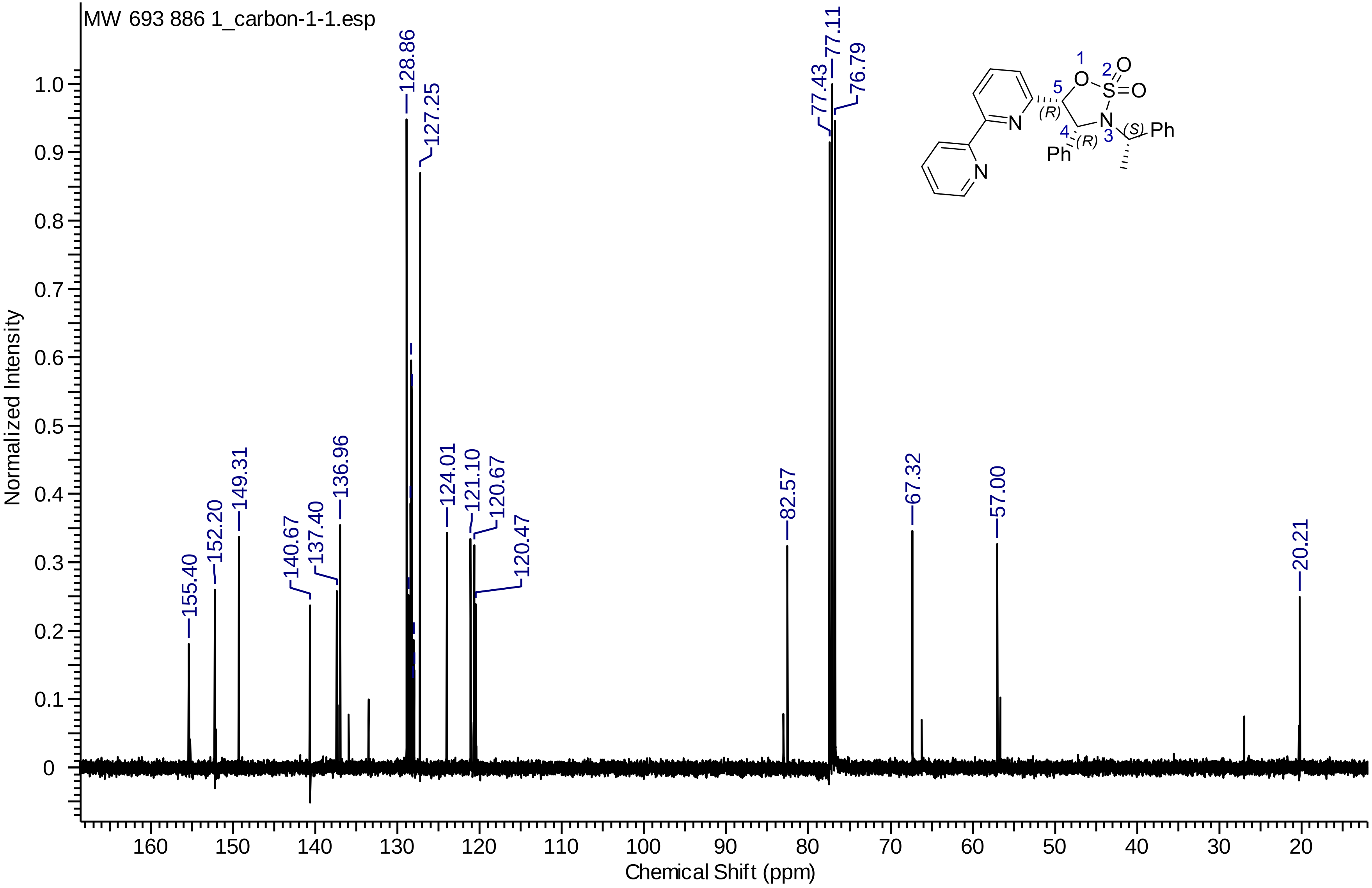

Figure S4. ^{13}C NMR spectrum (101 MHz, $CDCl_3$) for (4*R*,5*R*,1'*S*)-**14**

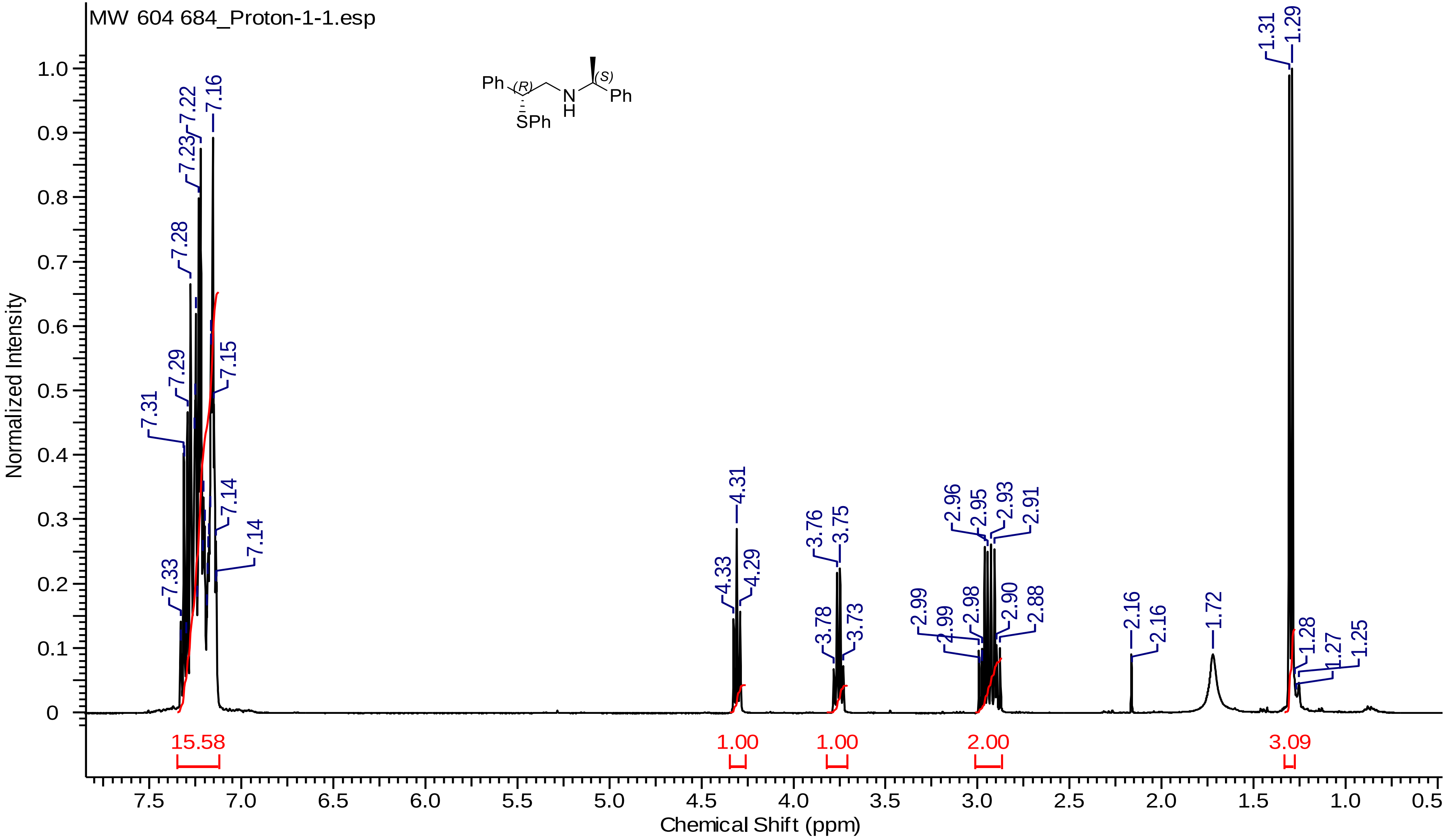

Figure S5. ^{1}H NMR spectrum (400 MHz, $CDCl_3$) for (2*R*,1'*S*)-**15**

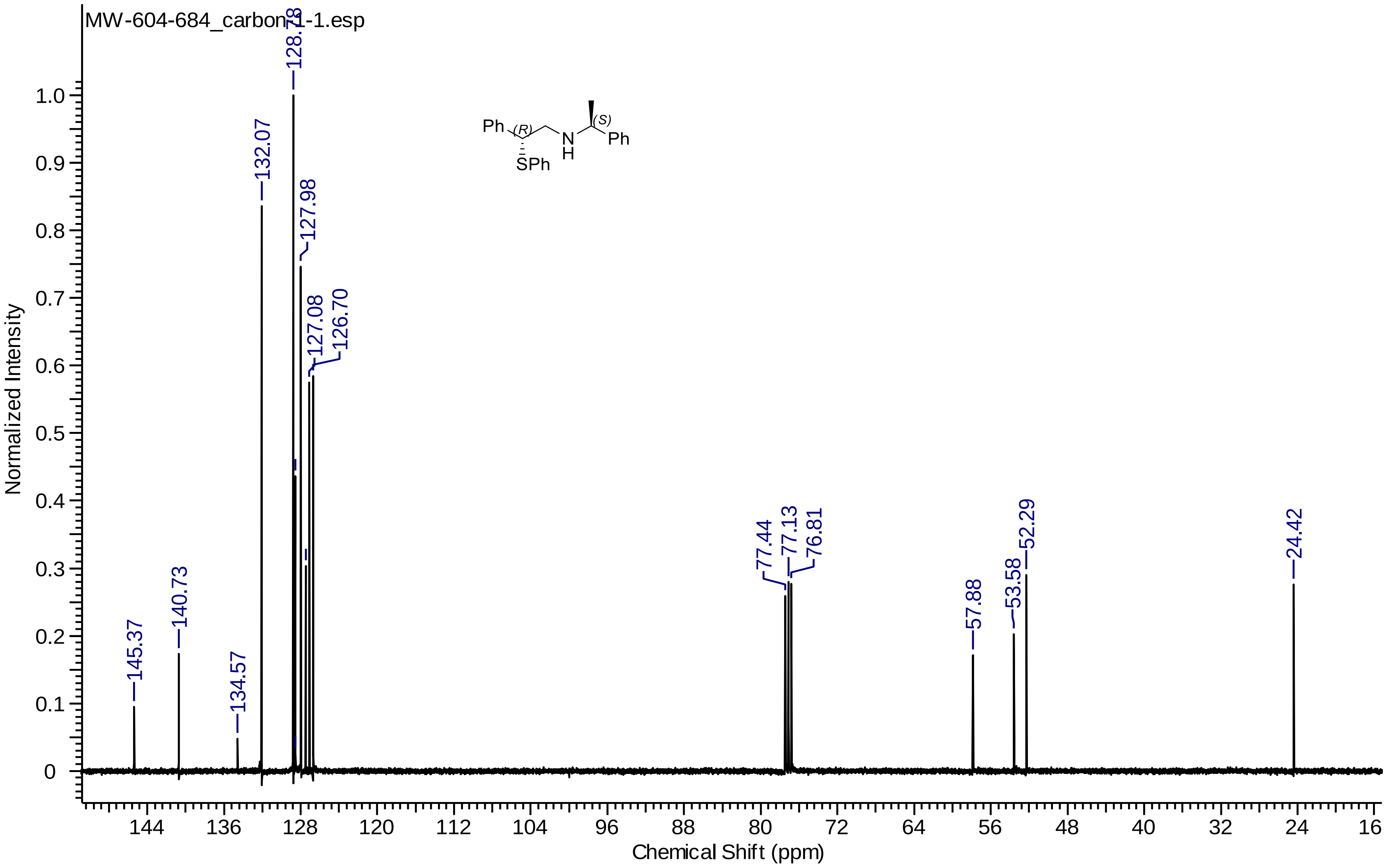

Figure S6. ^{13}C NMR spectrum (101 MHz, $CDCl_3$) for (2*R*,1'*S*)-**15**

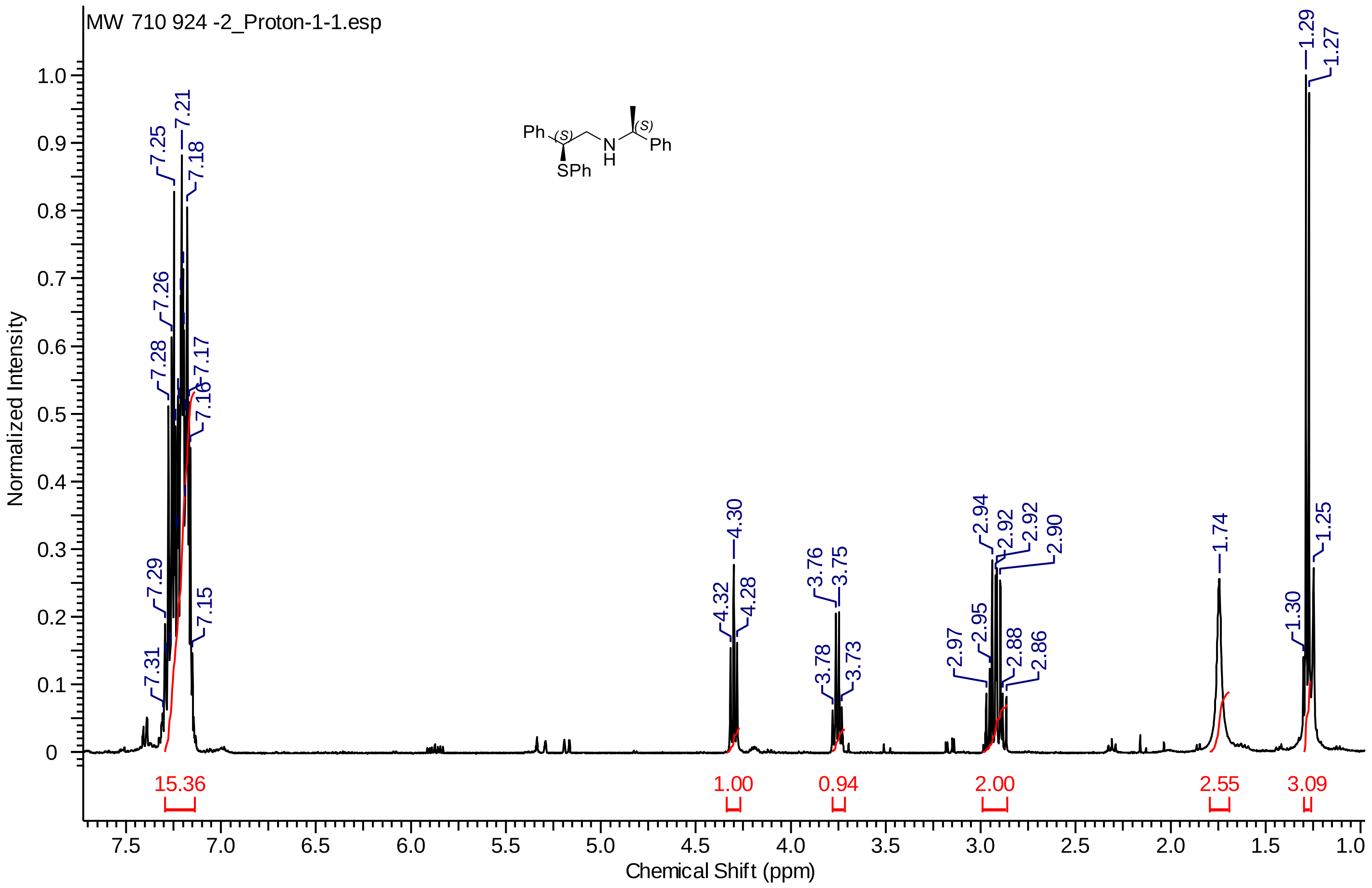

Figure S7. ^{13}C NMR spectrum (101 MHz, $CDCl_3$) for (2*S*,1'*S*)-**15**

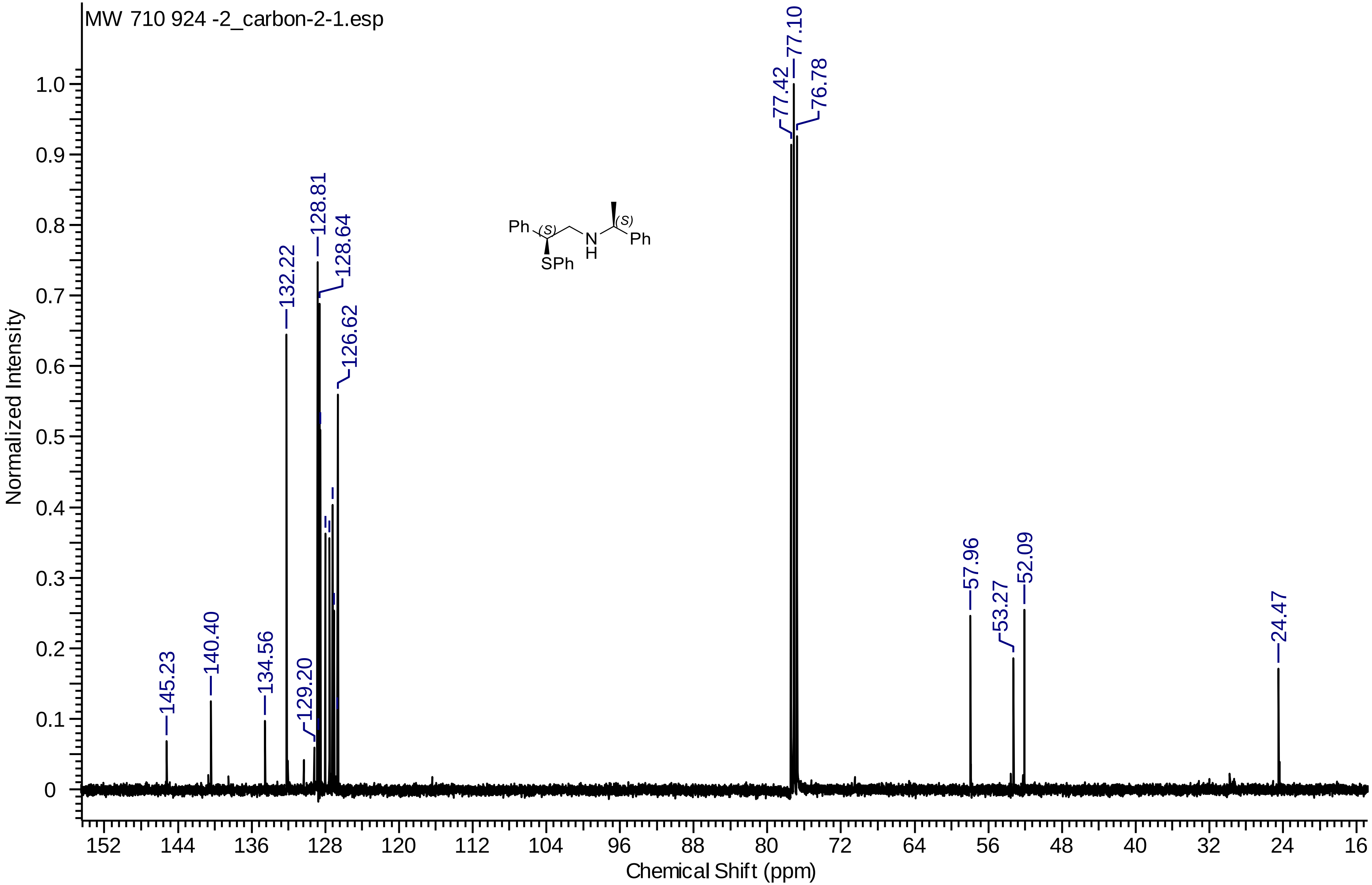

Figure S8. ^{13}C NMR spectrum (101 MHz, $CDCl_3$) for (2*S*,1'*S*)-**15**

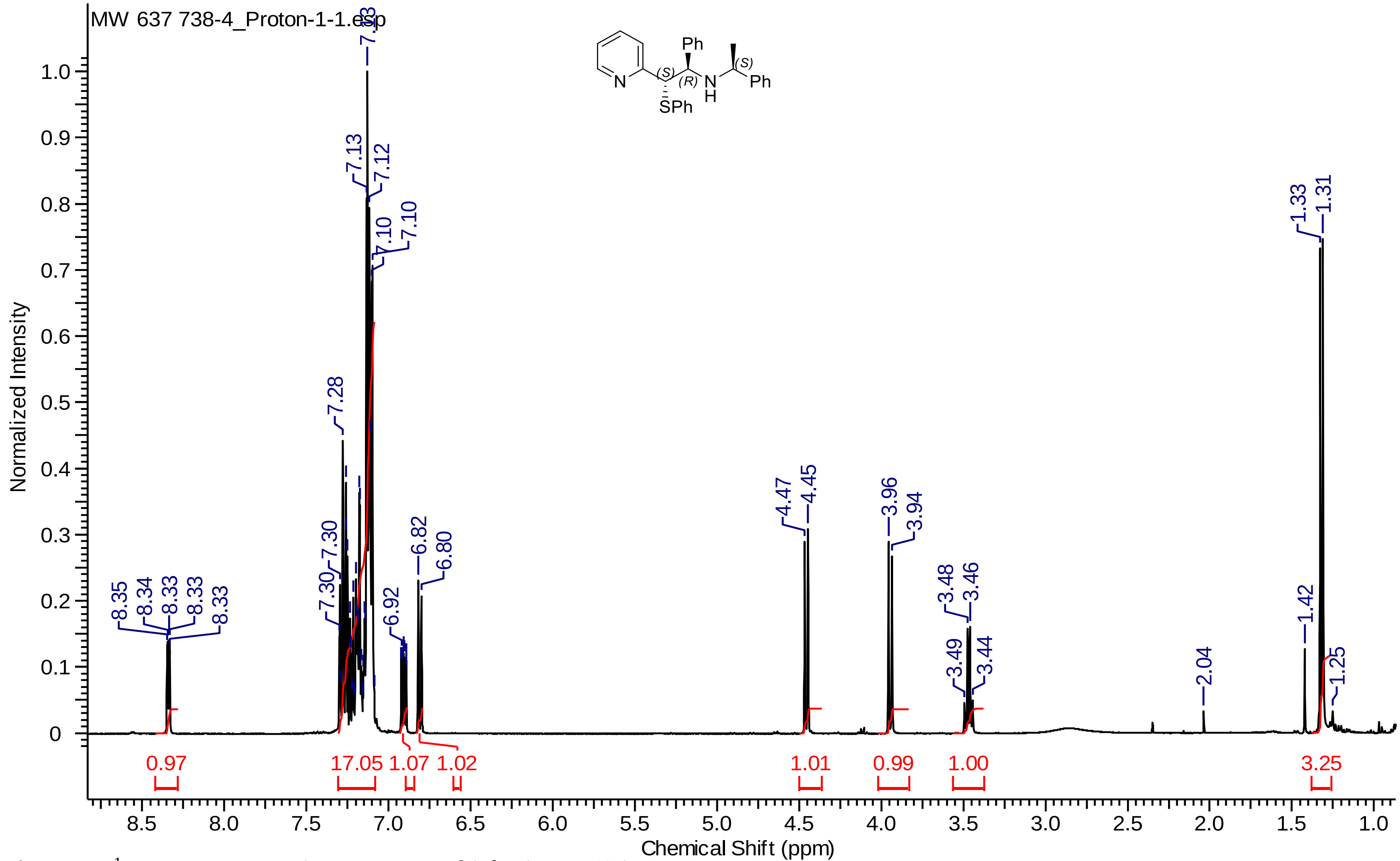

Figure S9. ^{1}H NMR spectrum (400 MHz, $CDCl_3$) for (1*R*,2*S*,1'*S*)-**16**

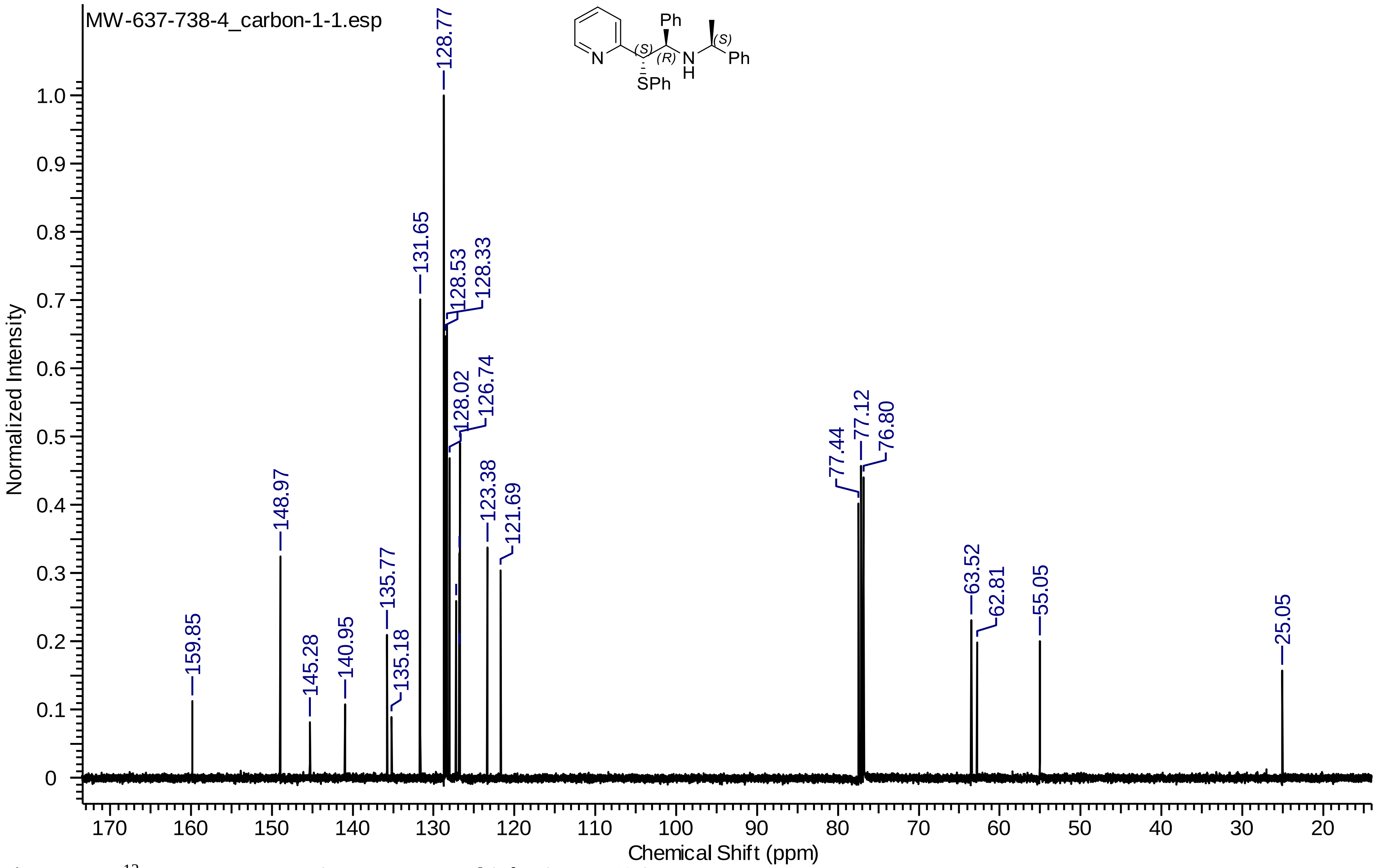

Figure S10. ^{13}C NMR spectrum (101 MHz, $CDCl_3$) for (1*R*,2*S*,1'*S*)-**16**

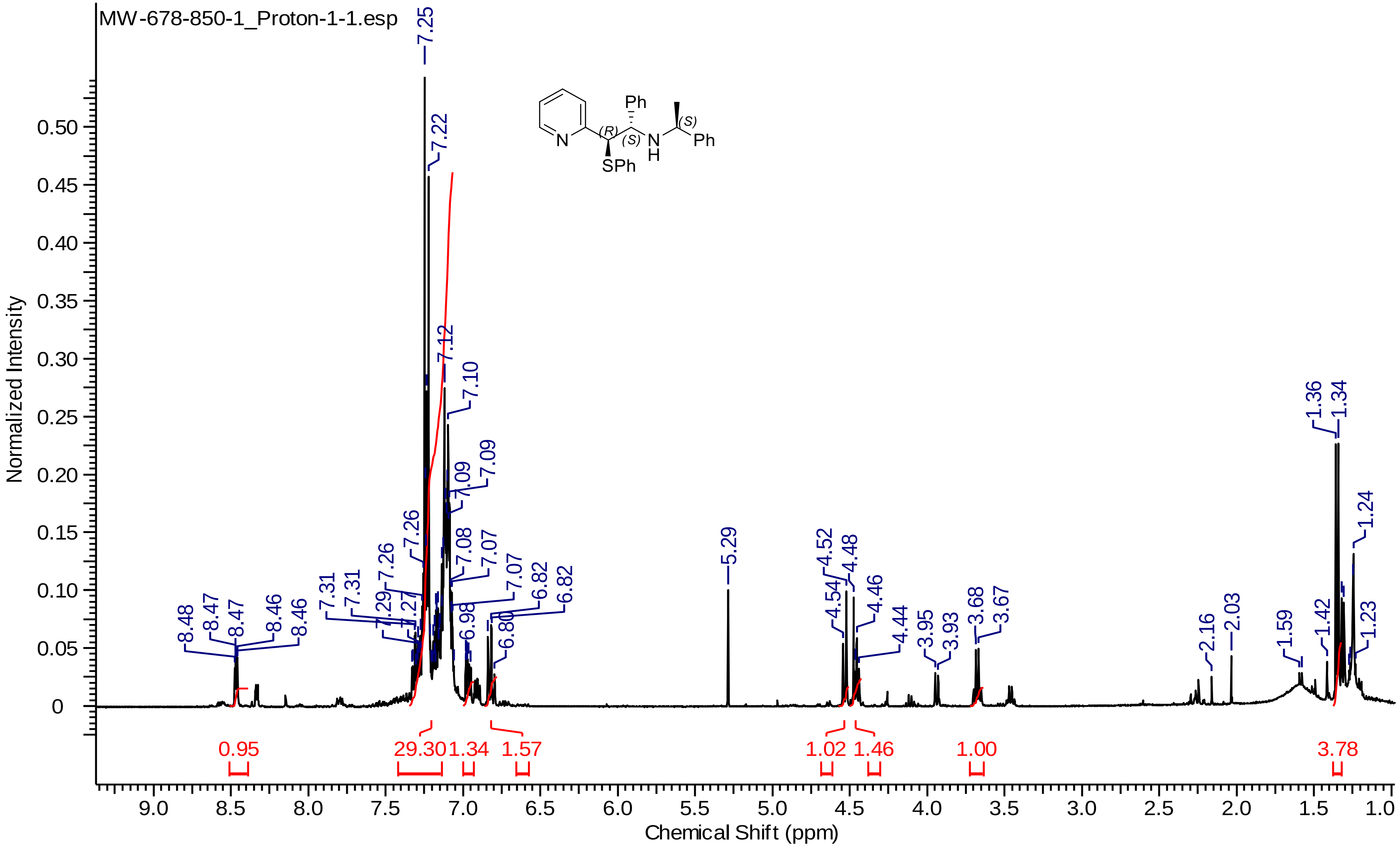

Figure S11. ^{1}H NMR spectrum (400 MHz, $CDCl_3$) for (1*S*,2*R*,1'*S*)-**16**

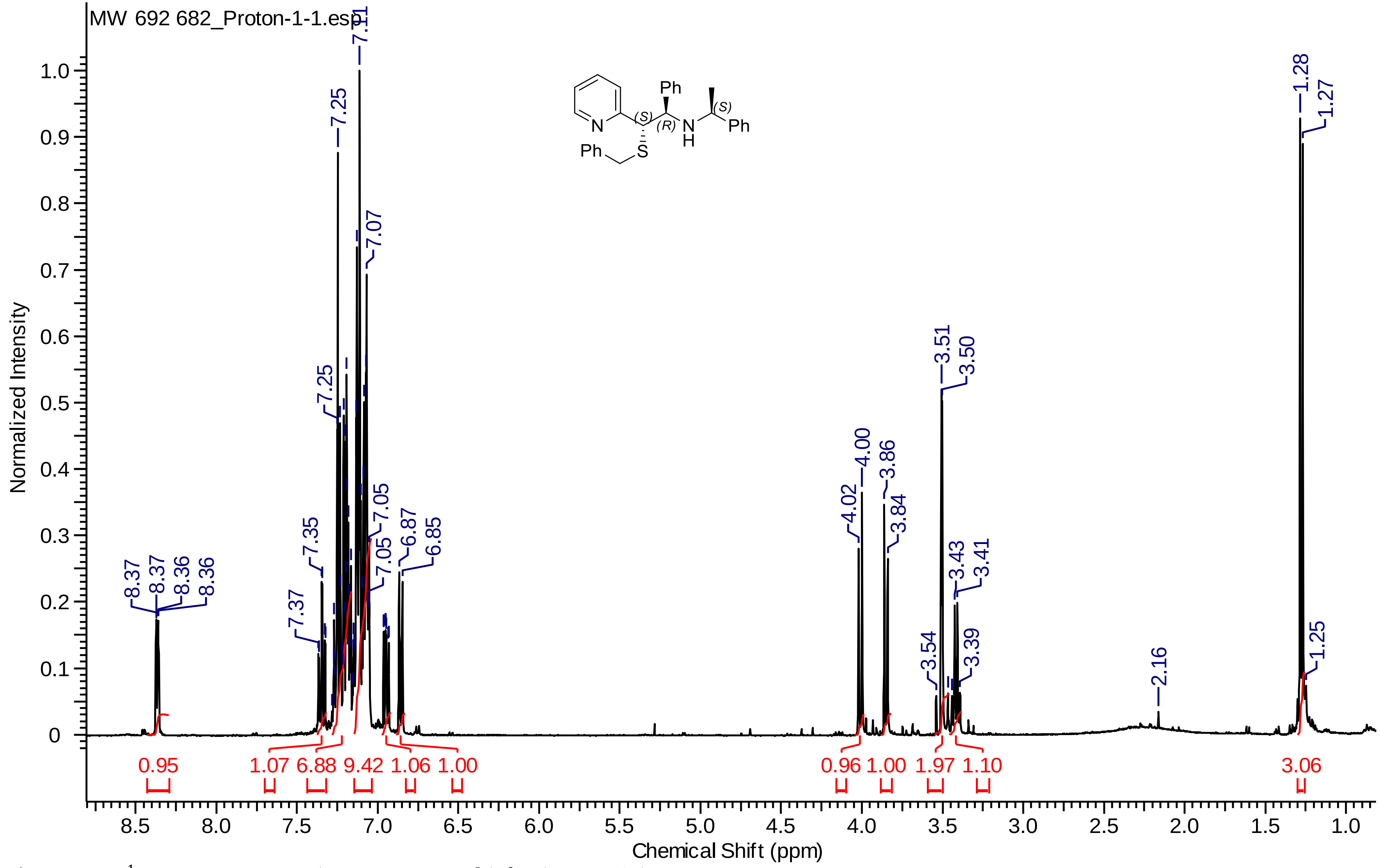

Figure S12. ^{1}H NMR spectrum (400 MHz, $CDCl_3$) for (1*R*,2*S*,1'*S*)-**17**

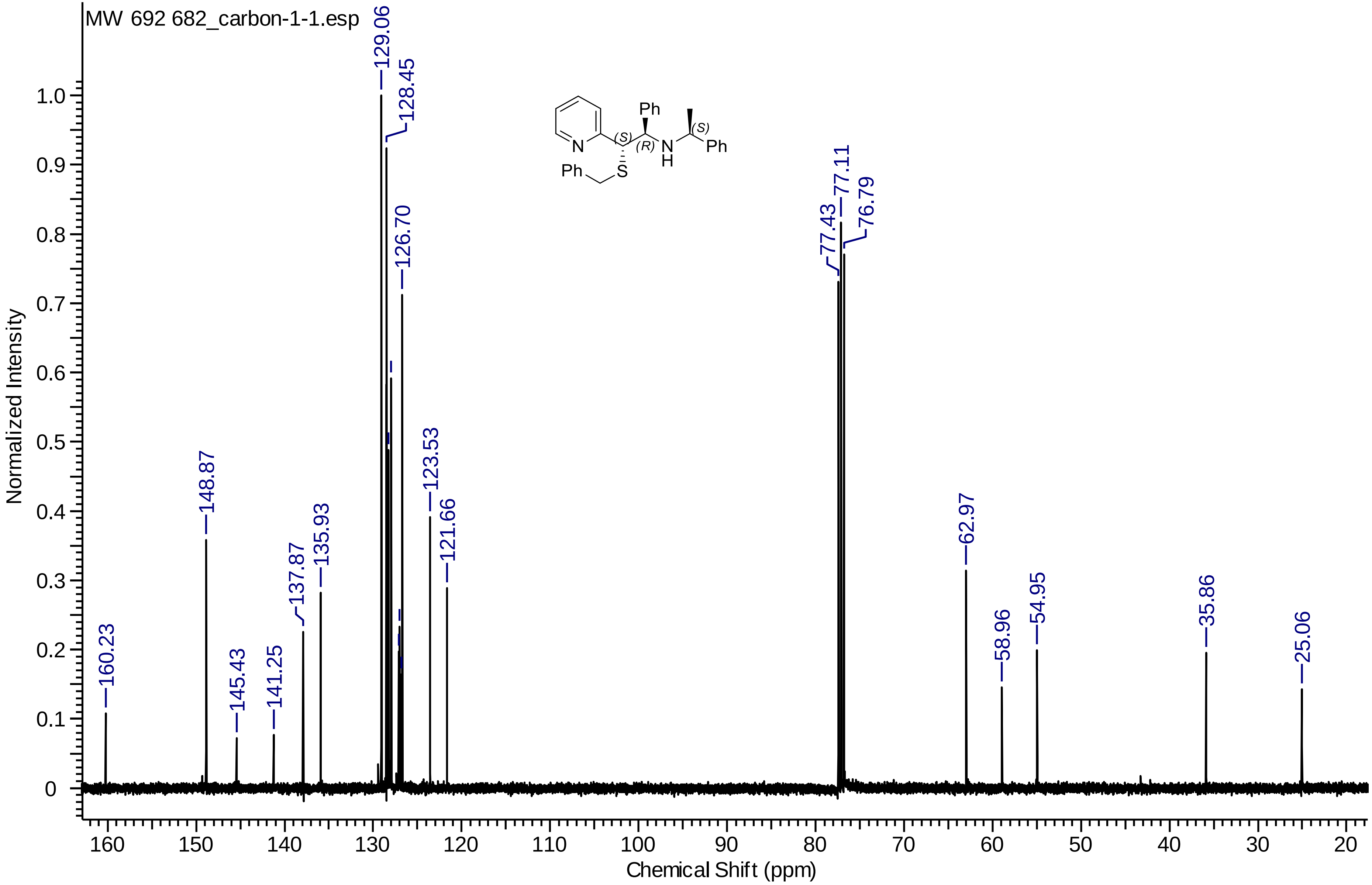

Figure S13. ^{13}C NMR spectrum (101 MHz, $CDCl_3$) for (1*R*,2*S*,1'*S*)-**17**

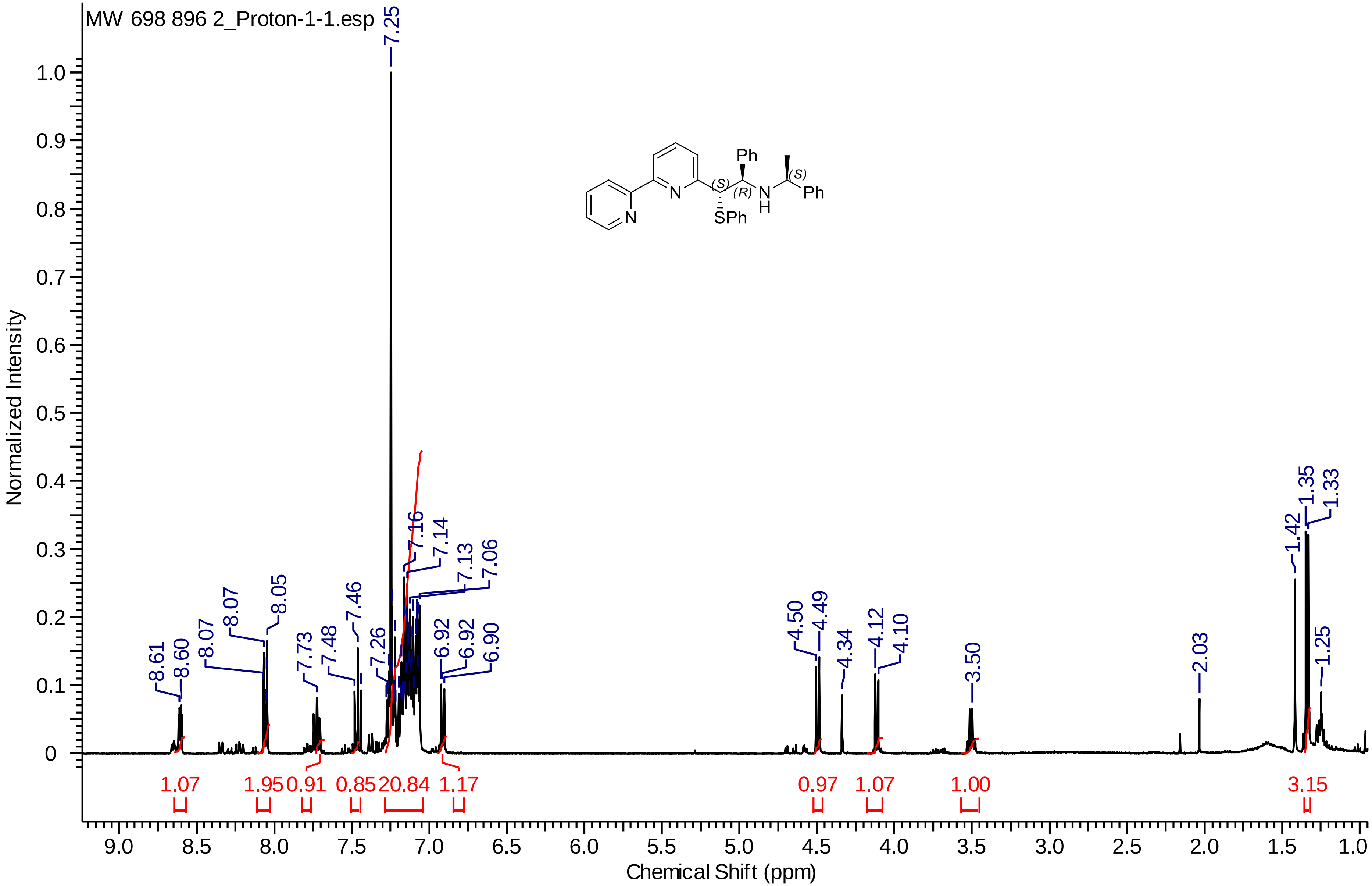

Figure S14. ^{1}H NMR spectrum (400 MHz, $CDCl_3$) for (1*R*,2*S*,1'*S*)-**18**

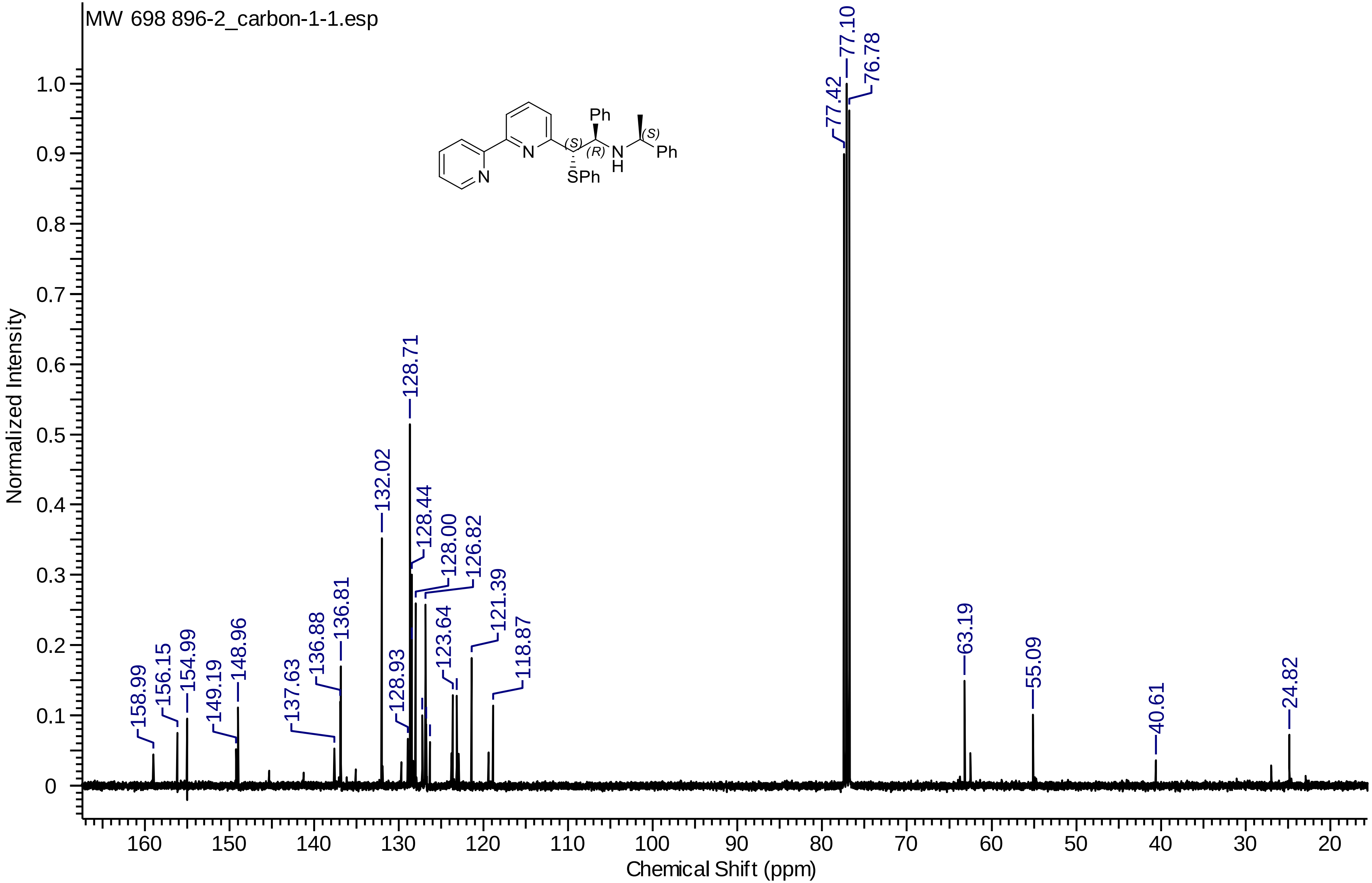

Figure S15. ^{13}C NMR spectrum (101 MHz, $CDCl_3$) for (1*R*,2*S*,1'*S*)-**18**

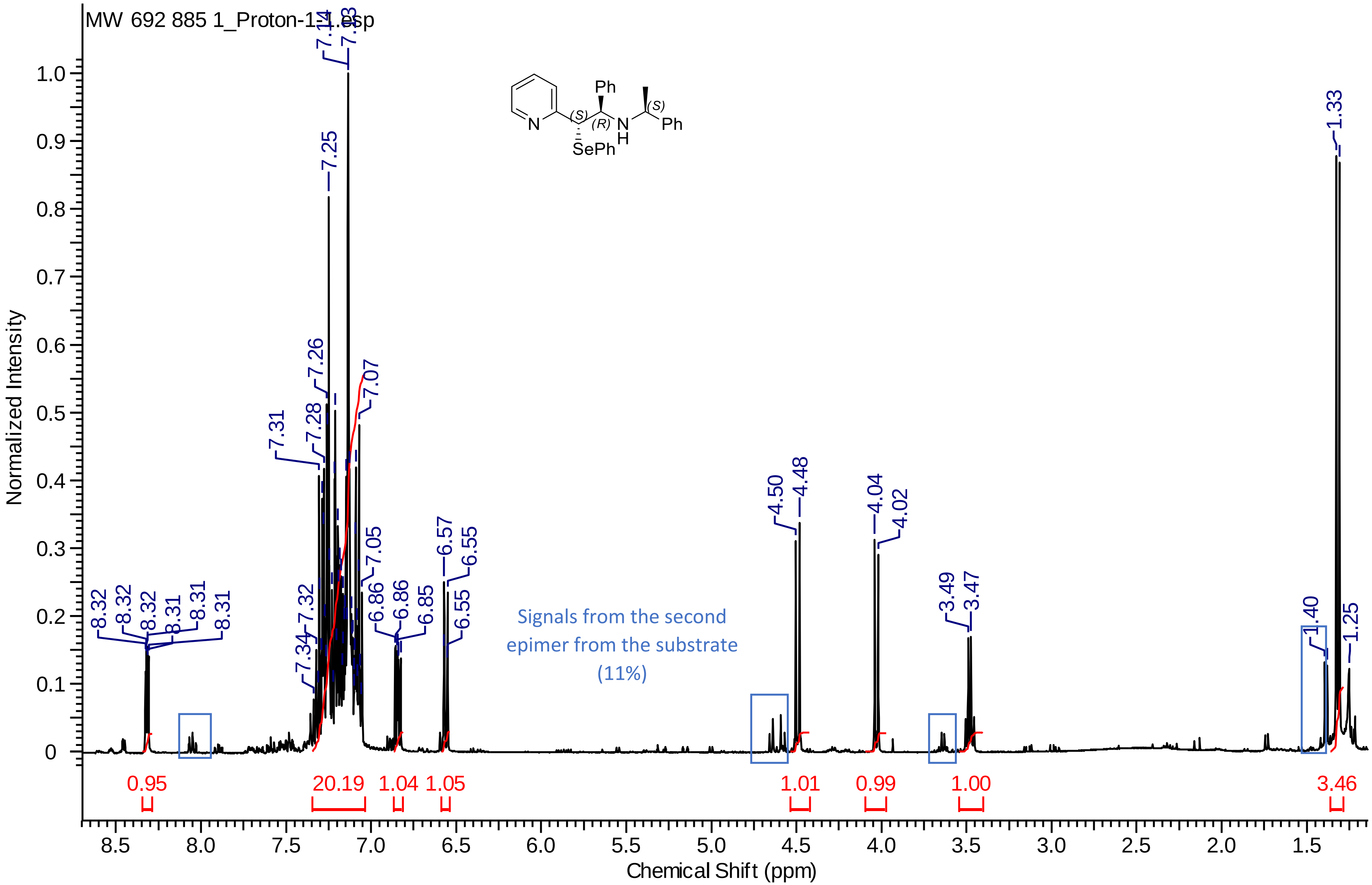

Figure S16. ^{1}H NMR spectrum (400 MHz, $CDCl_3$) for (1*R*,2*S*,1'*S*)-**19**

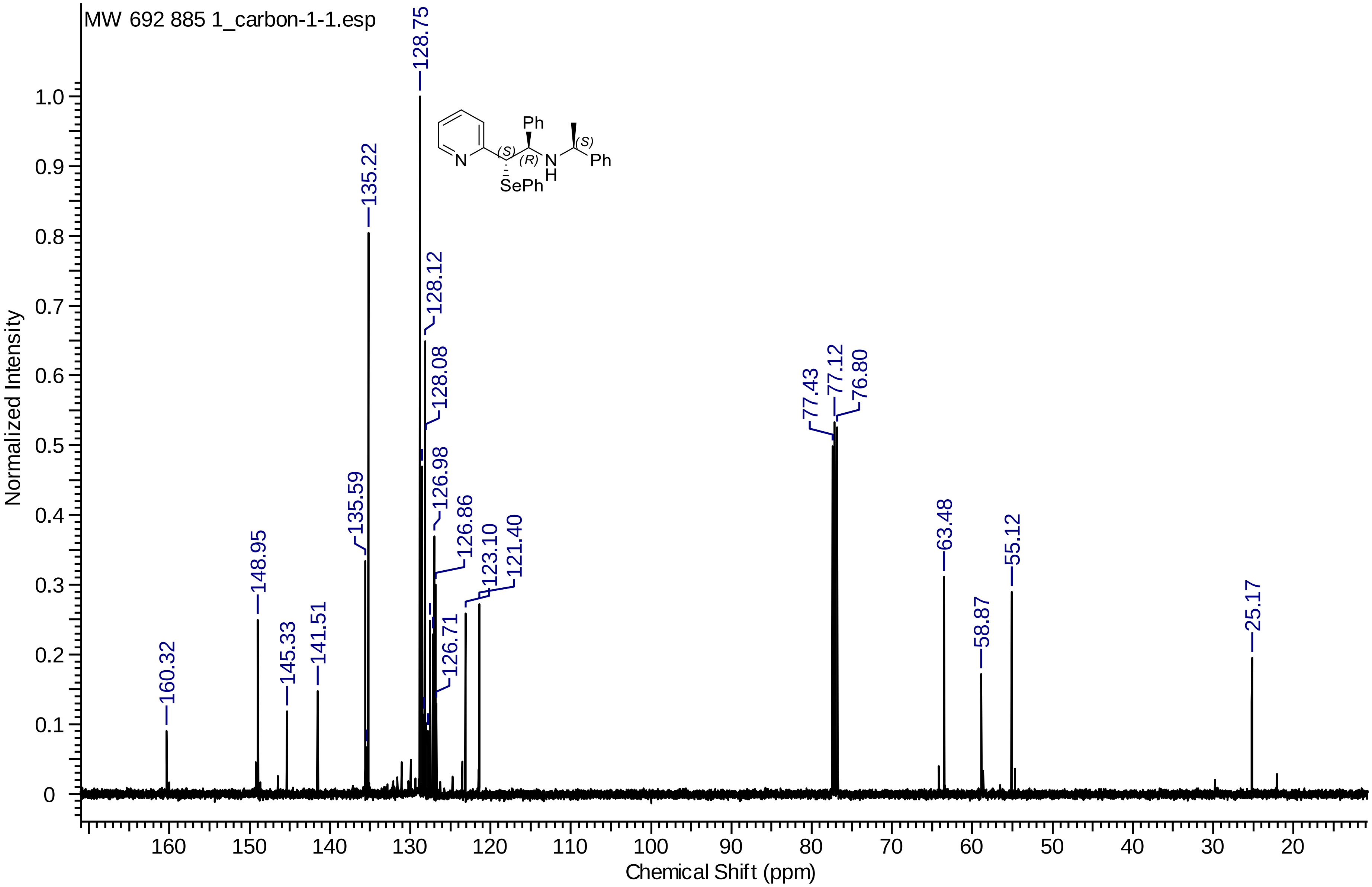

Figure S17. ^{13}C NMR spectrum (101 MHz, $CDCl_3$) for (1*R*,2*S*,1'*S*)-**19**

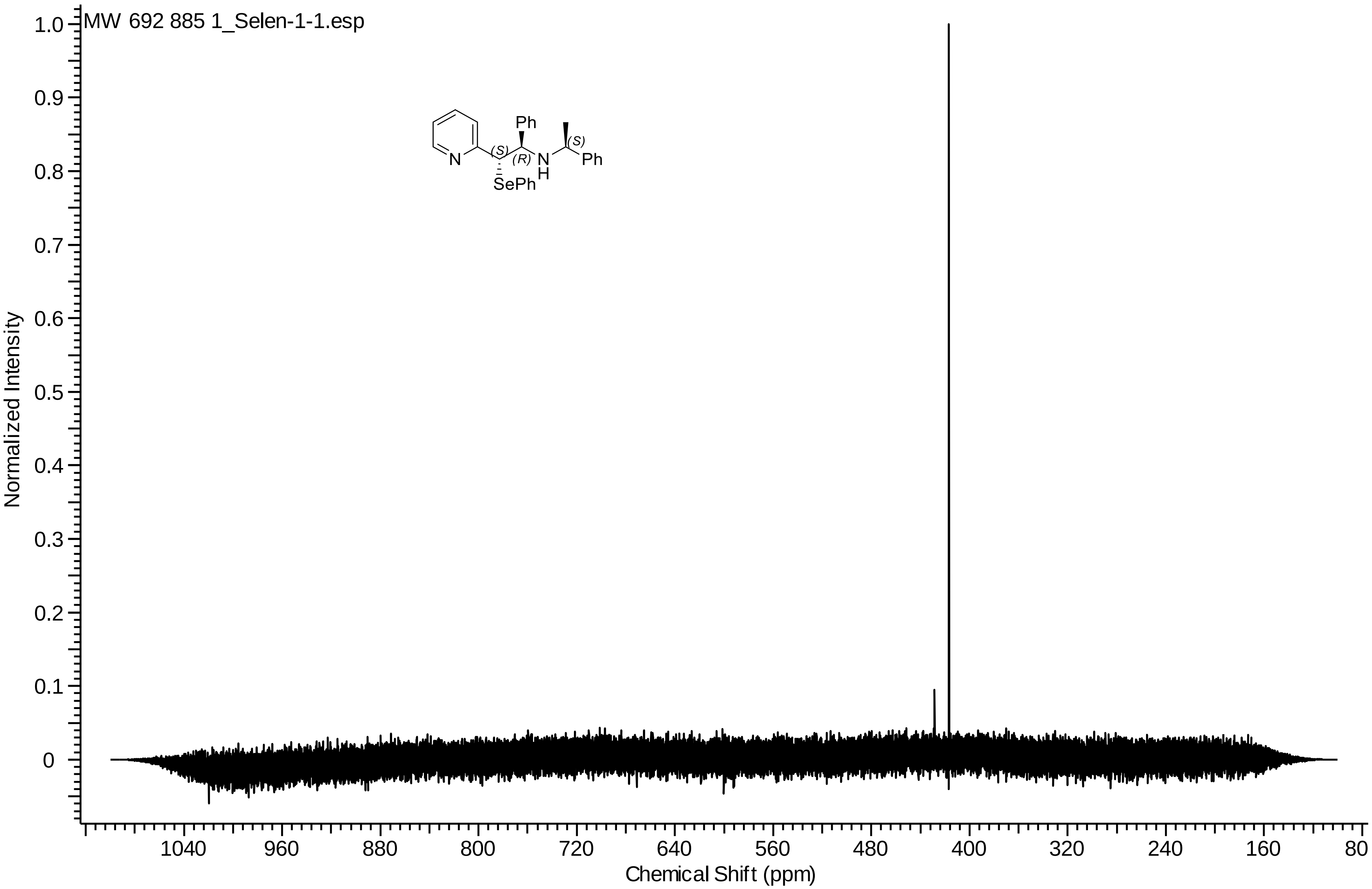

Figure S18. ^{77}Se NMR (38 MHz, $CDCl_3$): for (1*R*,2*S*,1'*S*)-**19**

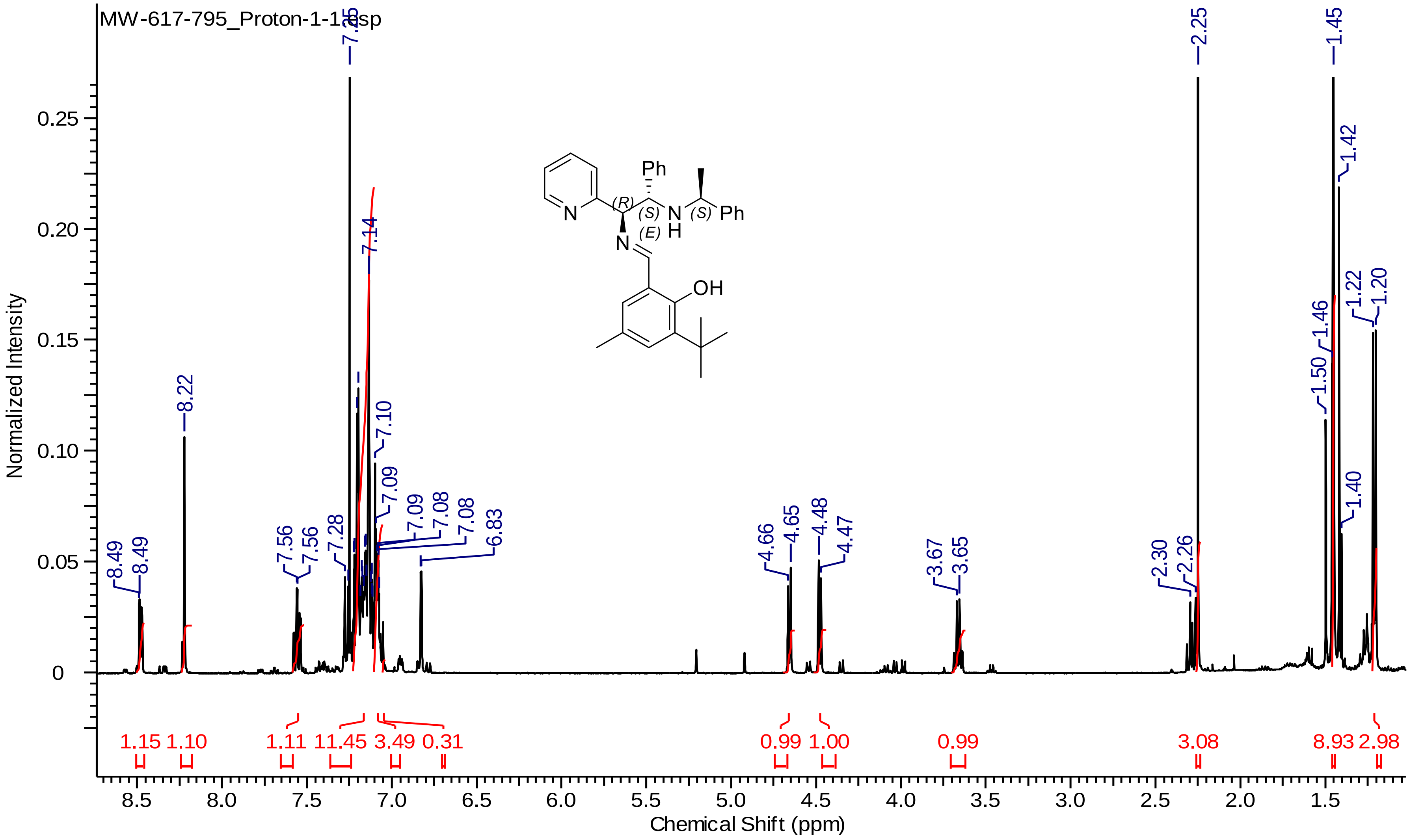

Figure S19. ^{1}H NMR spectrum (400 MHz, $CDCl_3$) for (1*S*,2*R*,1'*S*)-**22**

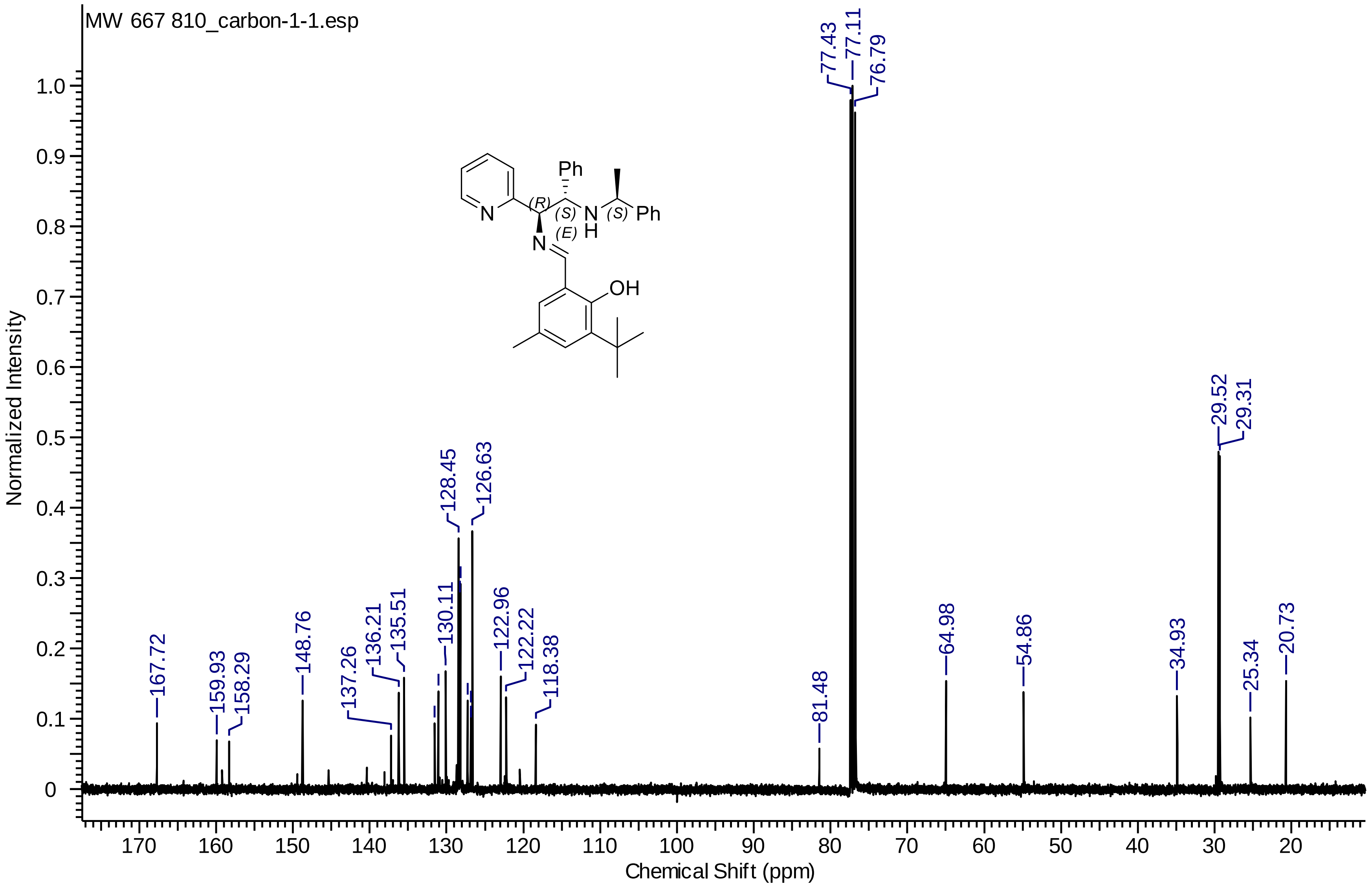

Figure S20. ^{13}C NMR spectrum (101 MHz, $CDCl_3$) for (1*S*,2*R*,1'*S*)-**22**

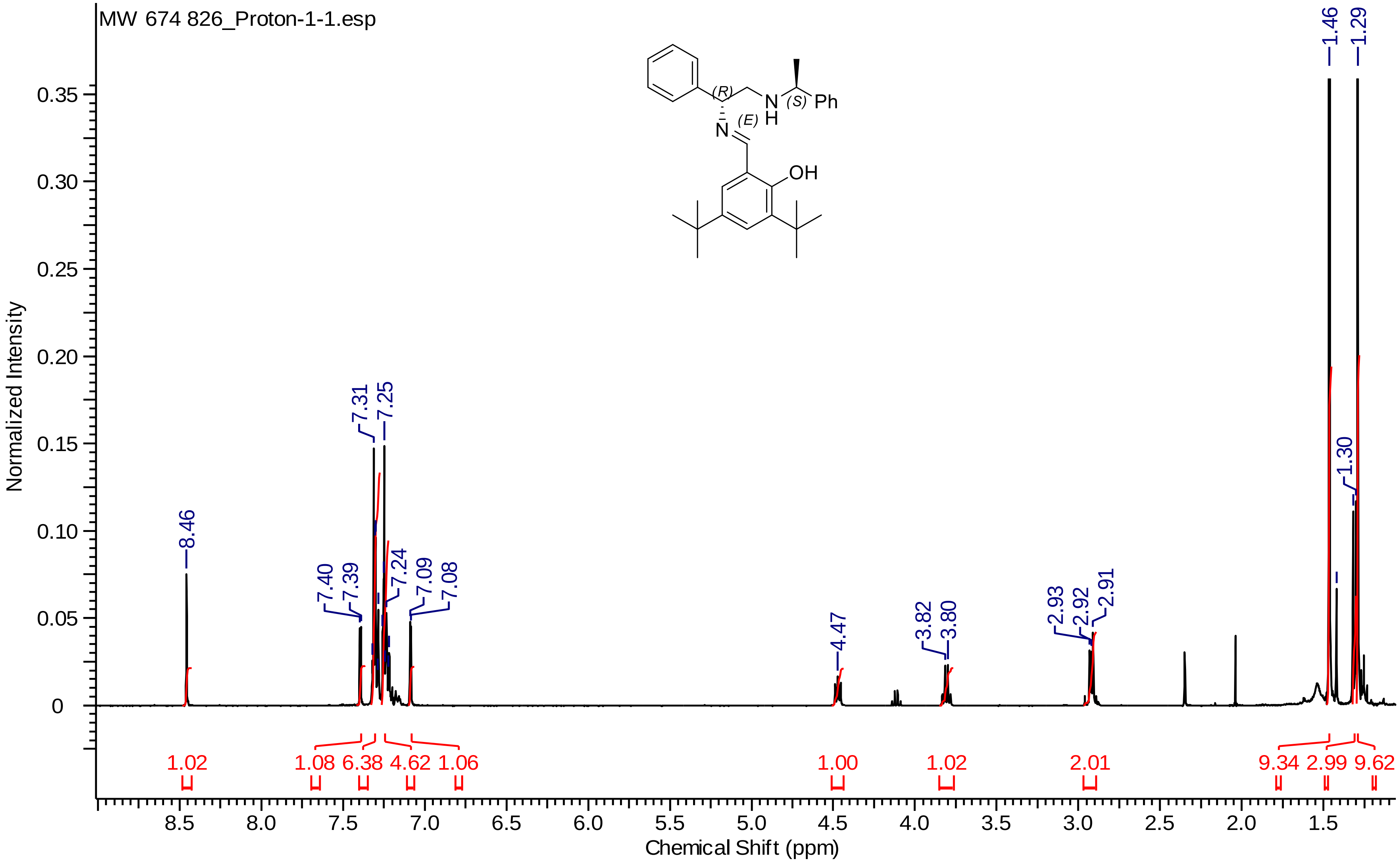

Figure S21. ^{1}H NMR spectrum (400 MHz, $CDCl_3$) for (2*R*,1'*S*)-**23**

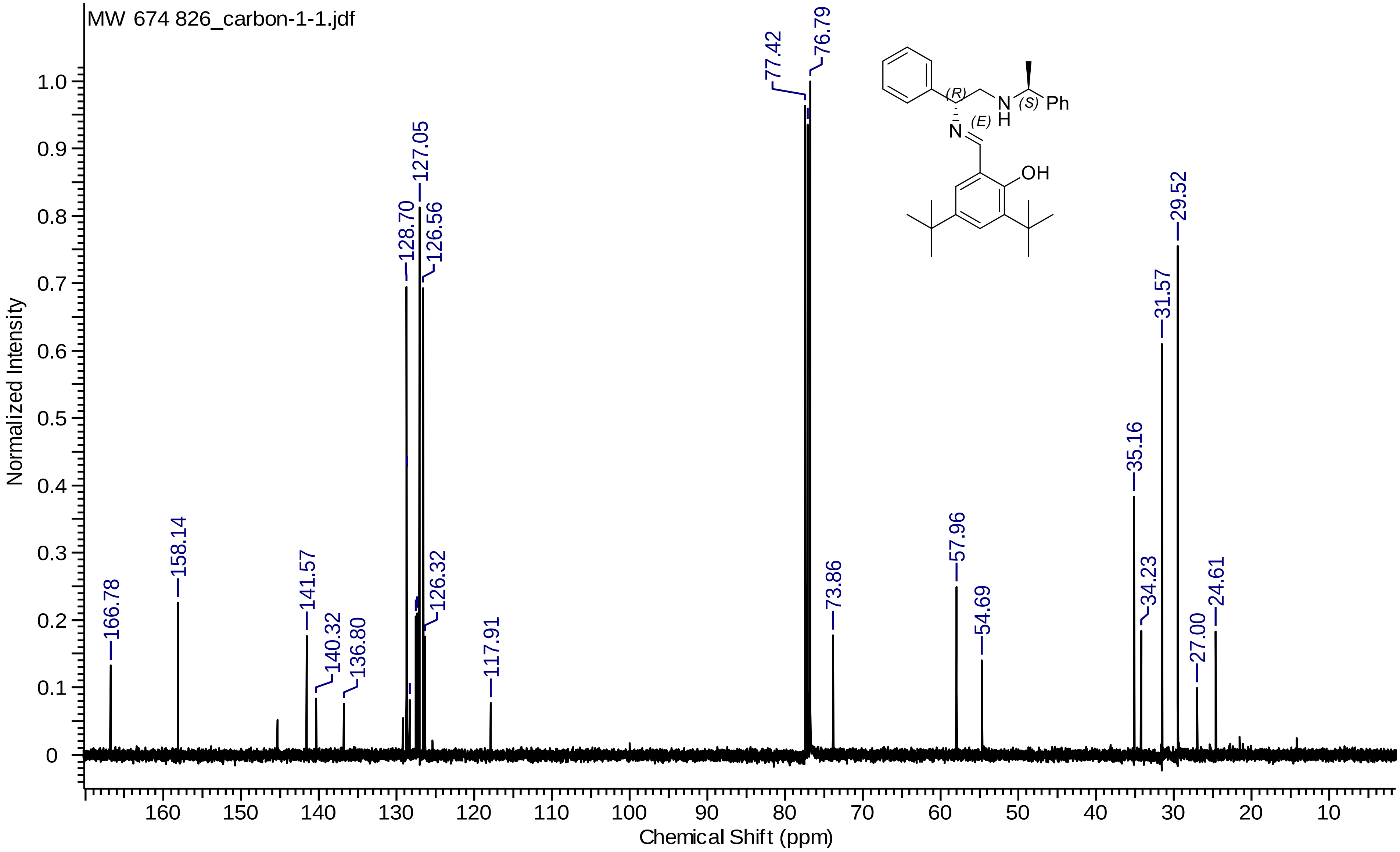

Figure S22. ^{13}C NMR spectrum (101 MHz, $CDCl_3$) for (2*R*,1'*S*)-**23**

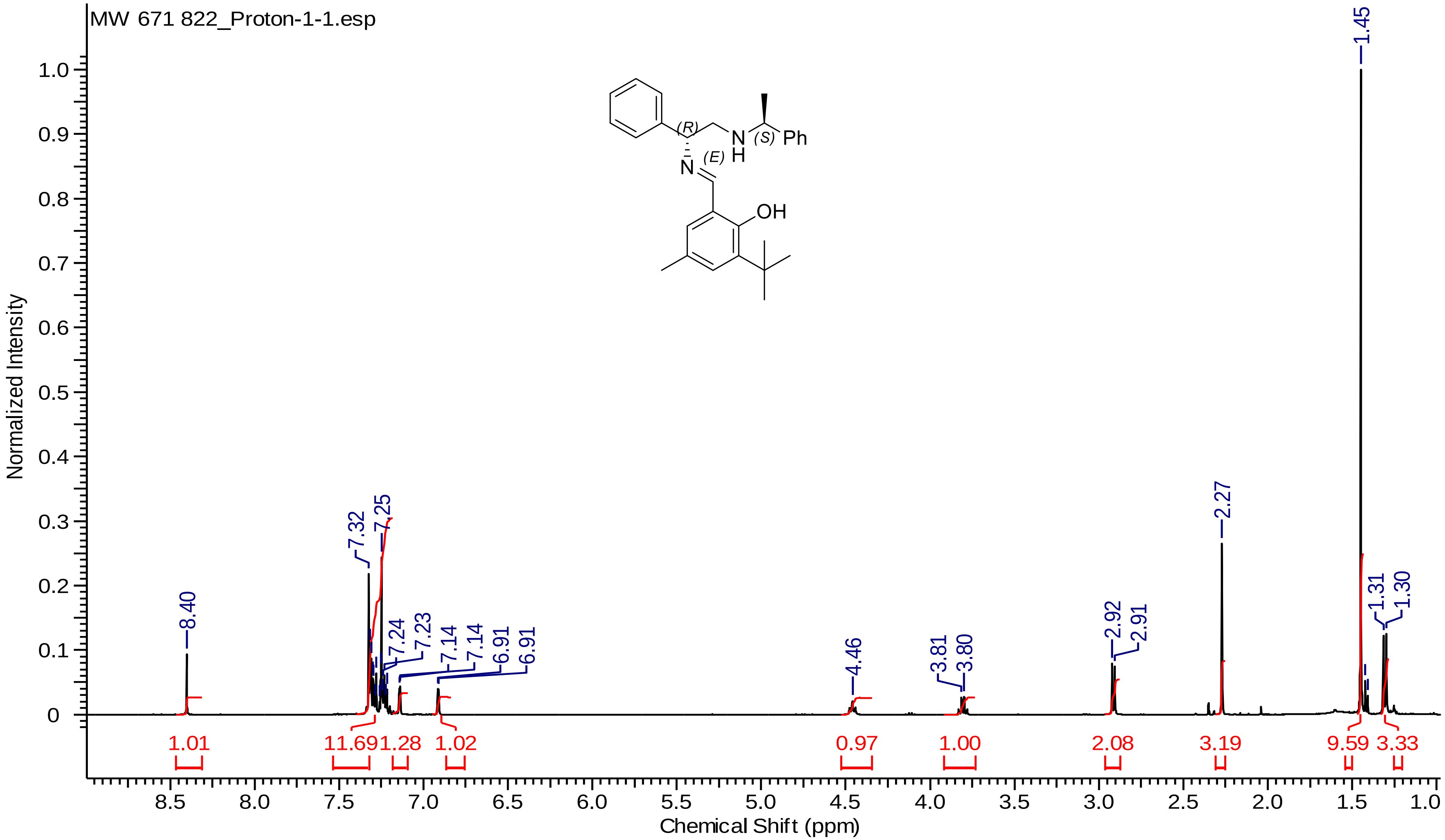

Figure S23. ^{1}H NMR spectrum (400 MHz, $CDCl_3$) for (2*R*,1'*S*)-**24**

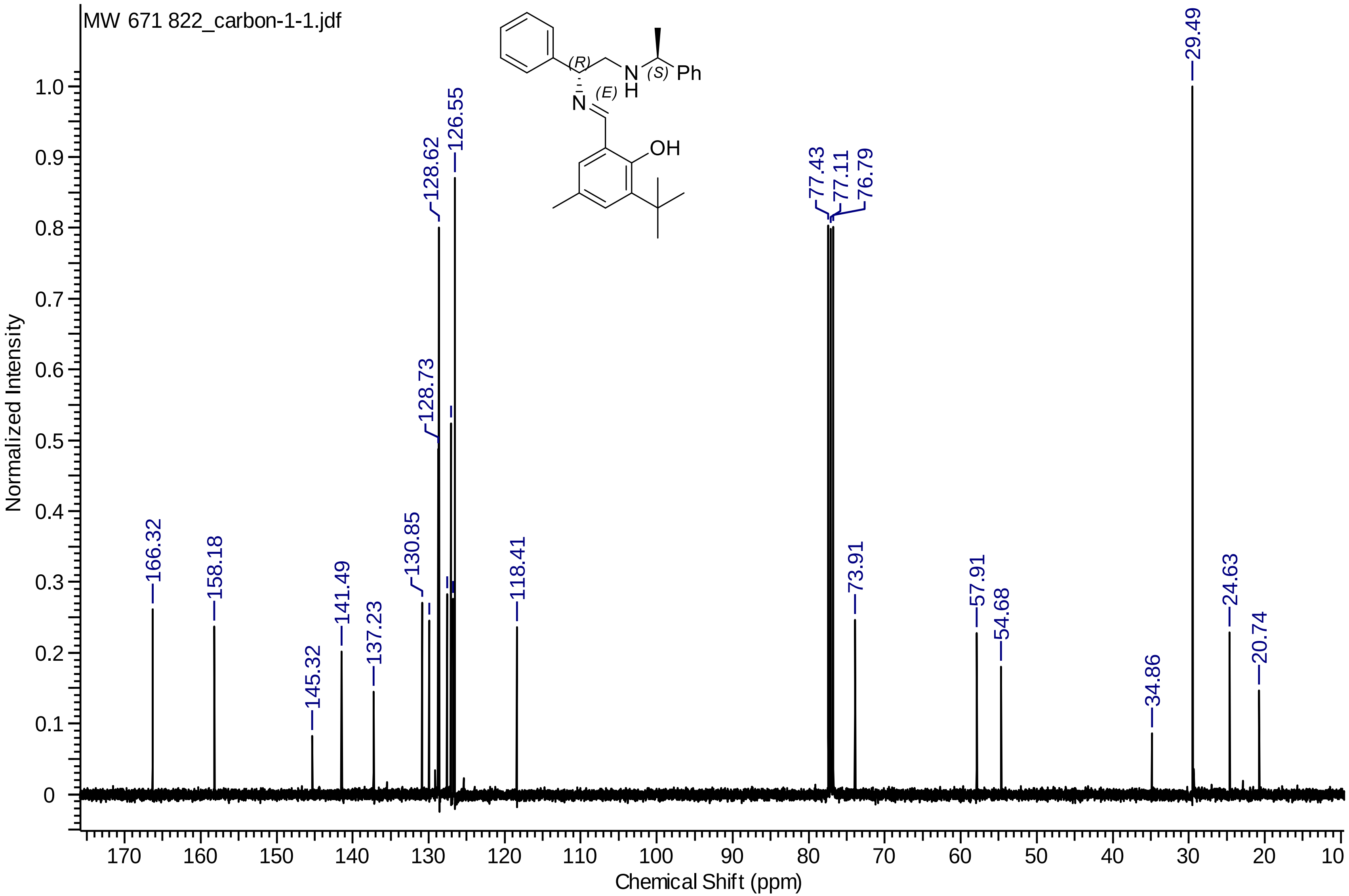

Figure S24. ^{13}C NMR spectrum (101 MHz, $CDCl_3$) for (2*R*,1’*S*)-**24**

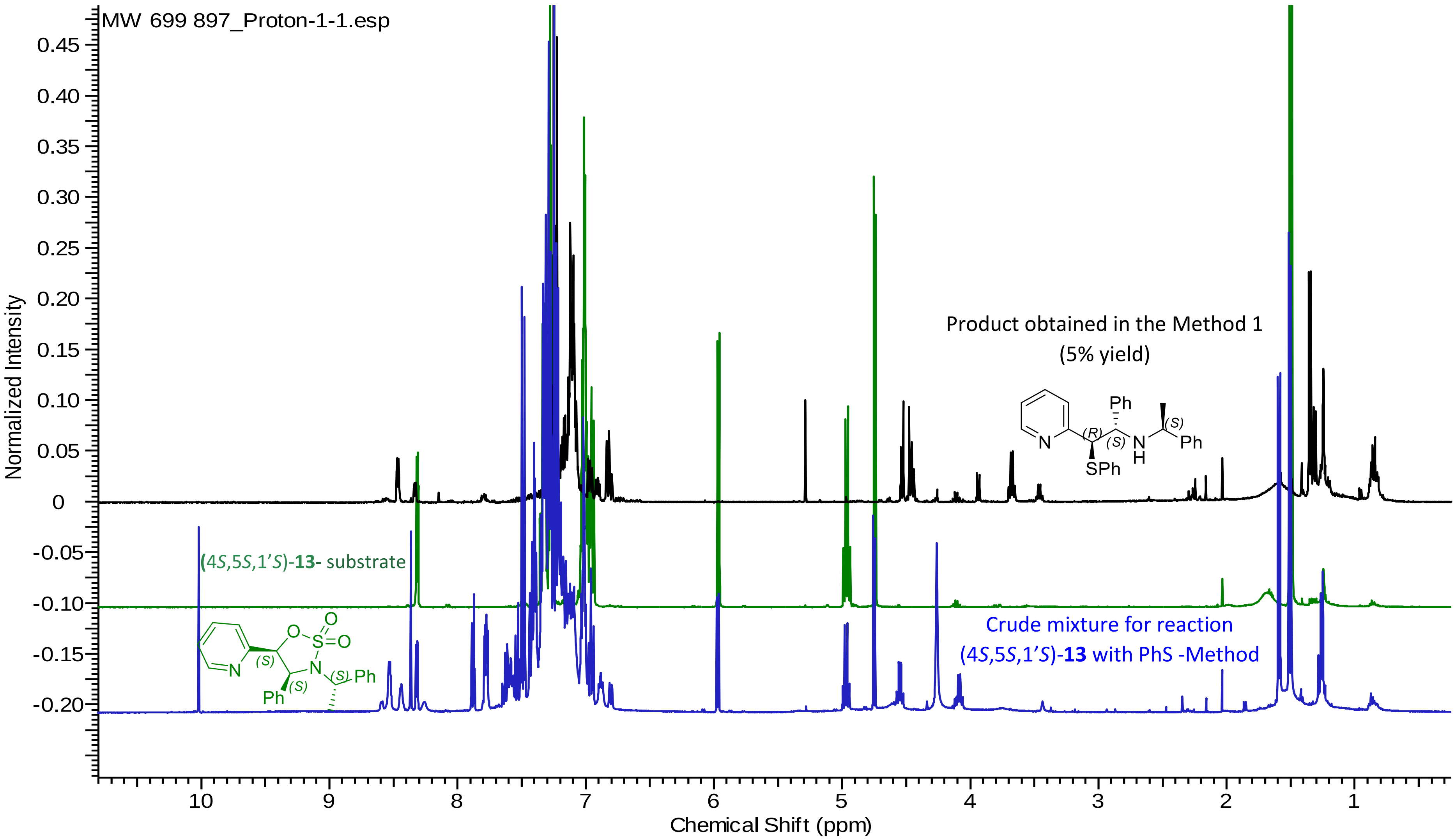

Figure S25. ^{1}H NMR spectra (400 MHz, $CDCl_3$) for crude mixture for reaction (4*S*,5*S*,1'*S*)-**13** with PhS -Method 2 (blue), substrate (green) and product (1*S*,2*R*,1'*S*)-**16** from Method 1 (black)

1. HPLC data

Alkylation of of *rac*-1,3-diphenyl-2-propenyl acetate (Enantiomeric excess was determined using a Chiralpak AD-H column (n-hexane/isopropanol 90/10, 1.0 mL/min, 254 nm)

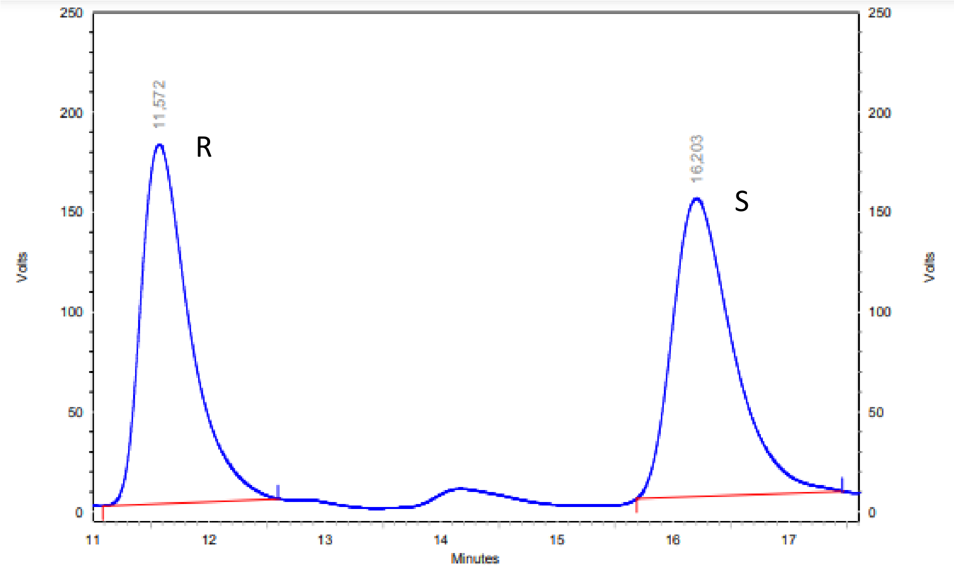

Retention Time	Area	Area %	Height	Height %
11,572	5207213	49,34	179548	54,64
16,203	5347142	50,66	149060	45,36
Totals	10554355	100,00	328608	100,00

Figure S26. HPLC chromatogram for *rac*-dimethyl [(2*E*)-1,3-diphenylprop-2-en-1-yl]propanedioate

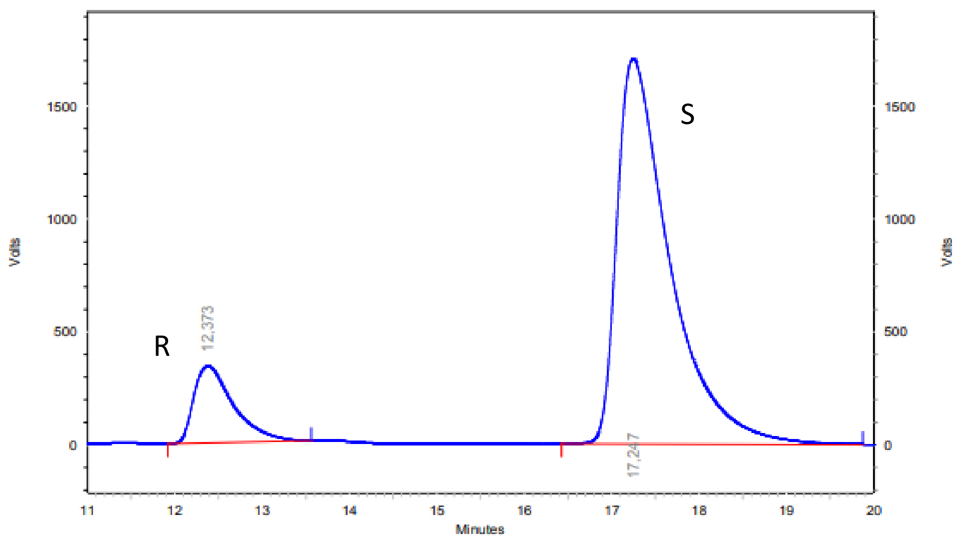

Retention Time	Area	Area %	Height	Height %
12,373	10642595	12,95	341391	16,69
17,247	71540398	87,05	1704525	83,31
Totals	82182993	100,00	2045916	100,00

Figure S27. HPLC chromatogram for dimethyl [(2*E*)-1,3-diphenylprop-2-en-1-yl]propanedioate obtained with catalyst (1*R*,2*S*,1’*S*)-**16**

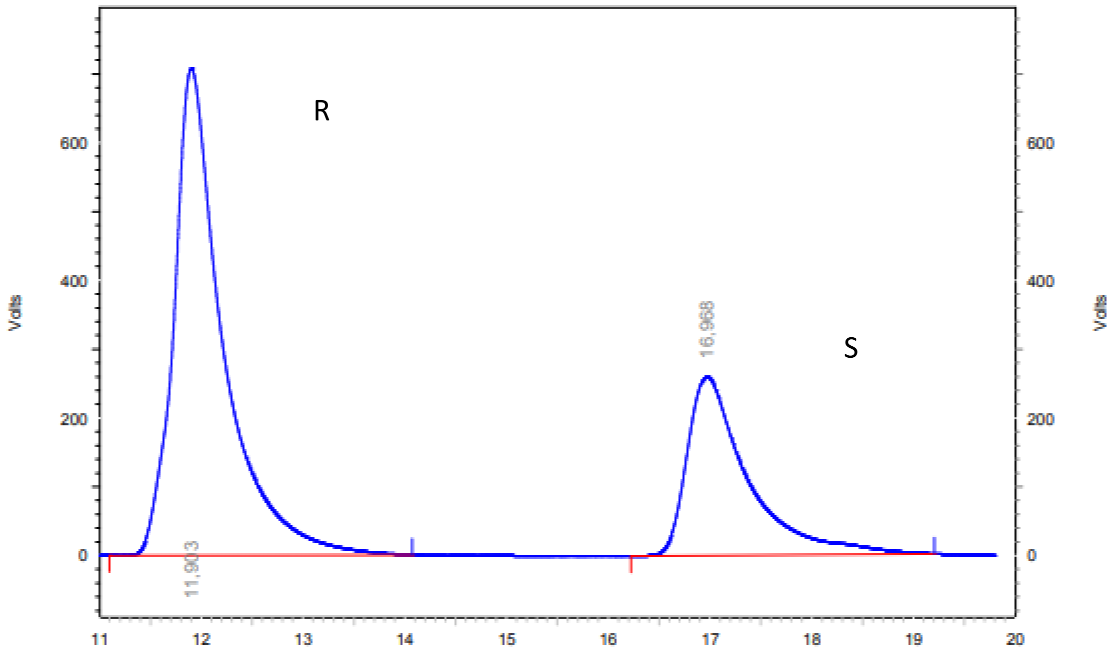

Retention Time	Area	Area %	Height	Height %
11,903	23749434	68,89	707689	73,15
16,968	10723569	31,11	259714	26,85
Totals	34473003	100,00	967403	100,00

Figure S28. HPLC chromatogram for dimethyl [(2*E*)-1,3-diphenylprop-2-en-1-yl]propanedioate obtained with catalyst (2*R*,1'*S*)**-24**

2. DFT calculation

Table S1. Comparison of experimental and DFT calculated ^{1}H NMR chemical shifts (GIAO DFT/mPW1PW91/6-311+G(2d,p)) for (1*R*, 2*S*,1'*S*)-**16** and (1*S*, 2*R*,1'*S*)-**16**

**Sygnał**	(1*R*, 2*S*,1'*S*)-**16**		(1S, 2R,1'*S*)-**16**	
	DFT δ, ppm	Experiment δ, ppm	DFT δ, ppm	Experiment δ, ppm
H-2	4.37	4.46	5,15	4,54
H-3	4.49	3.95	4,43	4,48
1-CH	3.63	3.46	4,49	3,68
CH_3	1.44	1.33	1,54	1,36
